# Embryonic transcriptome and proteome analyses on hepatic lipid metabolism in chickens divergently selected for abdominal fat content

**DOI:** 10.1186/s12864-018-4776-9

**Published:** 2018-05-23

**Authors:** Wei Na, Yuan-Yuan Wu, Peng-Fei Gong, Chun-Yan Wu, Bo-Han Cheng, Yu-Xiang Wang, Ning Wang, Zhi-Qiang Du, Hui Li

**Affiliations:** Key Laboratory of Chicken Genetics and Breeding, Ministry of Agriculture, Key Laboratory of Animal Genetics, Breeding and Reproduction, Education Department of Heilongjiang Province, College of Animal Science and Technology, Northeast Agricultural University, Harbin, 150030 People’s Republic of China

**Keywords:** Chicken, Embryo, Liver, Lipid metabolism, Digital gene expression, Proteomics

## Abstract

**Background:**

In avian species, liver is the main site of de novo lipogenesis, and hepatic lipid metabolism relates closely to adipose fat deposition. Using our fat and lean chicken lines of striking differences in abdominal fat content, post-hatch lipid metabolism in both liver and adipose tissues has been studied extensively. However, whether molecular discrepancy for hepatic lipid metabolism exists in chicken embryos remains obscure.

**Results:**

We performed transcriptome and proteome profiling on chicken livers at five embryonic stages (E7, E12, E14, E17 and E21) between the fat and lean chicken lines. At each stage, 521, 141, 882, 979 and 169 differentially expressed genes were found by the digital gene expression, respectively, which were significantly enriched in the metabolic, PPAR signaling and fatty acid metabolism pathways. Quantitative proteomics analysis found 20 differentially expressed proteins related to lipid metabolism, PPAR signaling, fat digestion and absorption, and oxidative phosphorylation pathways. Combined analysis showed that genes and proteins related to lipid transport (intestinal fatty acid-binding protein, nucleoside diphosphate kinase, and apolipoprotein A-I), lipid clearance (heat shock protein beta-1) and energy metabolism (NADH dehydrogenase [ubiquinone] 1 beta subcomplex subunit 10 and succinate dehydrogenase flavoprotein subunit) were significantly differentially expressed between the two lines.

**Conclusions:**

For hepatic lipid metabolism at embryonic stages, molecular differences related to lipid transport, lipid clearance and energy metabolism exist between the fat and lean chicken lines, which might contribute to the striking differences of abdominal fat deposition at post-hatch stages.

**Electronic supplementary material:**

The online version of this article (10.1186/s12864-018-4776-9) contains supplementary material, which is available to authorized users.

## Background

Broiler is the most efficient meat-producing farm animal, and contributes to alleviating the challenge of food security imposed upon the human society [1, 2]. For over half a century, commercial broiler has been selected intensively for growth rate and feed efficiency [1]. However, intensive selection on fast growth rate brings along adverse outcomes, such as obesity and related metabolic syndromes [3]. Excessive fat deposition is undesirable, since it degrades meat quality, decreases feed efficiency, and increases production and health cost [4]. Currently, to reduce fat deposition is still a main objective of commercial broiler selection and breeding program [1, 5].

Unlike in mammals, chicken lipogenesis is very limited in the adipose tissue [6], and more than 70% of de novo fatty acid synthesis takes place in the liver instead [7]. Fatty acids synthesized in the liver are incorporated into triacylglycerols, and secreted as very low-density lipoprotein (VLDL). After hydrolysis by the lipoprotein lipase (LPL), fatty acids released from VLDL penetrate adipocytes, where they are re-esterified and stored as triglycerides [8]. Thus, accumulation of triacylglycerols in adipocytes is closely related to lipid metabolism in the liver [9].

The Northeast Agricultural University broiler lines (NEAUHLF) originate from a commercial Arbor Acres grandsire line, and are under divergent selection for abdominal fat content based on abdominal fat percentage (AFP) and plasma VLDL concentration since 1996 [10]. NEAUHLF is a unique animal model to study the molecular mechanism of adipose tissue growth and development. In previous studies, using adipose and liver tissues from the fat and lean chickens at various post-hatch stages (1, 4 and 7 weeks of age), we have found a number of differentially expressed genes (DEGs) and proteins (DEPs) related to lipid metabolism by the microarray and proteomics methods, such as peroxisome proliferator-activated receptor gamma (*PPARγ*), liver basic fatty acids binding protein (*LBFABP*), *LPL*, adipocyte fatty acid-binding protein (AFABP), apolipoprotein A-I (ApoA-I), and long-chain acyl-coenzyme A dehydrogenase (ACADL) [10–13].

Embryonic stage now occupies nearly one third of the time to market size for broilers, and is vital to the post-hatch performance of broilers. In order to see if molecular differences of hepatic lipid metabolism existed between our two chicken lines at embryonic stages, we performed digital gene expression profiling and quantitative proteomics on the livers of chicken embryos taken from five embryonic stages, embryonic day 7 (E7), E12, E14, E17, and day 1 after hatch (E21). We identified DEGs and DEPs associated with hepatic lipid metabolism at embryonic stages between the two chicken lines, which could help explain the striking differences of post-hatch abdominal fat content.

## Results

### Transcriptome analysis on embryonic livers

**Fig. 1:**
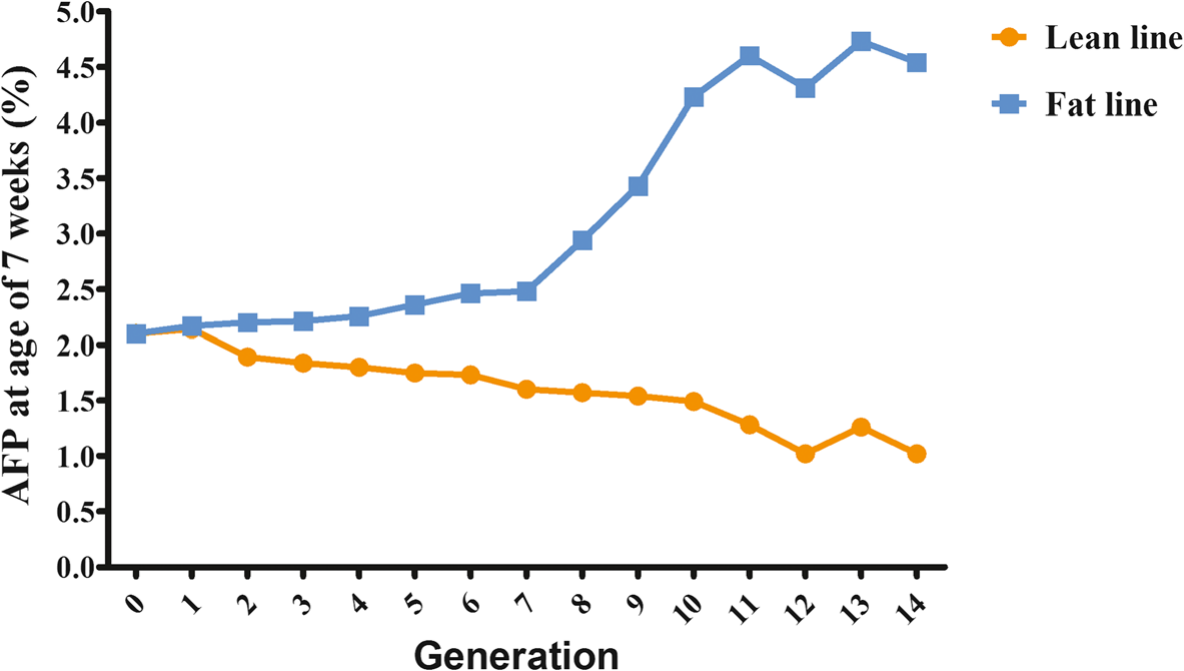
Divergent selection on abdominal fat percentage (AFP) at 7 weeks of age. Starting from generation 4, significant differences existed between the two chicken lines

Between our broiler lines divergently selected for AFP, significant differences exist since generation 4 (Fig. 1). From the fat and lean chicken lines at generation 14, which had 4.5-fold difference of AFP at 7 weeks of age [14], we collected hepatic tissues from embryos at five important developmental stages (E7, E12, E14, E17 and E21). Total RNAs isolated from hepatic tissues were submitted for sequencing by the digital gene expression profiling technology. For each of the 10 sequenced libraries, an average of 8500 genes (54.8% of all annotated protein-coding genes) was found, and a large number of novel transcripts were also detected (from 36,569 to 45,021) (Additional file 1).

**Fig. 2:**
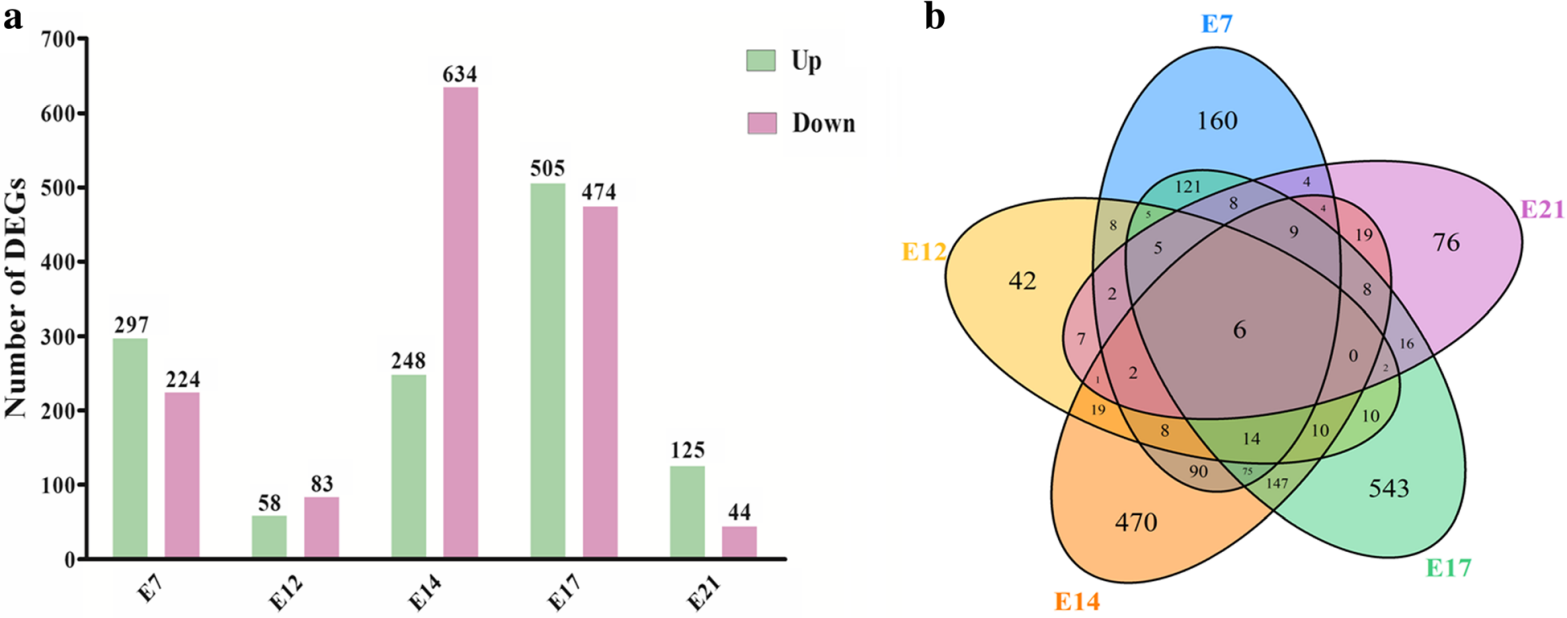
Differentially expressed genes (DEGs) identified for hepatic tissues in chicken embryos. **a** Number of DEGs at five developmental stages between the fat and lean lines. Up and Down, up- and down-regulated DEGs in the fat line; **b** Venn diagram for DEGs identified

To identify genes differentially expressed (false discovery rate ≤ 0.001 and fold changes ≥2) and potentially involved in hepatic lipid metabolism, we compared the gene expression profiles between the fat and lean chicken lines. We found 521, 141, 882, 979 and 169 DEGs (Additional files 2, 3, 4, 5 and 6) for the five embryonic stages, respectively (Fig. 2a). Furthermore, after analyzing the number of DEGs among the five different stages, we observed that E17 shared the largest number of DEGs with other embryonic stages (Fig. 2b). However, there were only 6 DEGs common to all five developmental stages, which were involved in cell metabolism, and cell apoptosis pathways, respectively (Fig. 2b and Table 1).

**Table 1:** Common DEGs to all five embryonic stages

Gene	Average fold-change (Log2 ratio)^a^	GO: Cell component	GO: Molecular function	GO: Biological processes
*HSPA9*	+ 2.58	mitochondrial matrix	protein binding	cellular protein metabolic process, protein targeting, negative regulation of apoptosis
*LOC777109*	+ 9.89	membrane, I band	cytoskeletal protein binding	striated muscle cell development
*LOC769366*	+ 9.30	NA	NA	NA
*BAP1*	−7.39	PcG protein complex	binding, thiolester hydrolase activity, small conjugating protein-specific protease activity	cell growth, cell cycle, modification-dependent protein catabolic process, cell proliferation
*XPO5*	−1.72	nuclear lumen	RNA binding	protein targeting, gene silencing
*ALDH7A1*	−1.40	intracellular membrane-bounded organelle, cytoplasmic part	aldehyde dehydrogenase [NAD(P)+] activity	cellular metabolic process, sensory perception of mechanical stimulus

*NA* not available. *HSPA9* heat shock 70 kDa protein 9, *BAP1* BRCA1 associated protein 1, *XPO5* exportin 5, *ALDH7A1* aldehyde dehydrogenase 7 family member A1

^a^gene expression fold-changes across the 5 embryonic stages; + and -, up- and down-regulated in the fat line

**Fig. 3:**
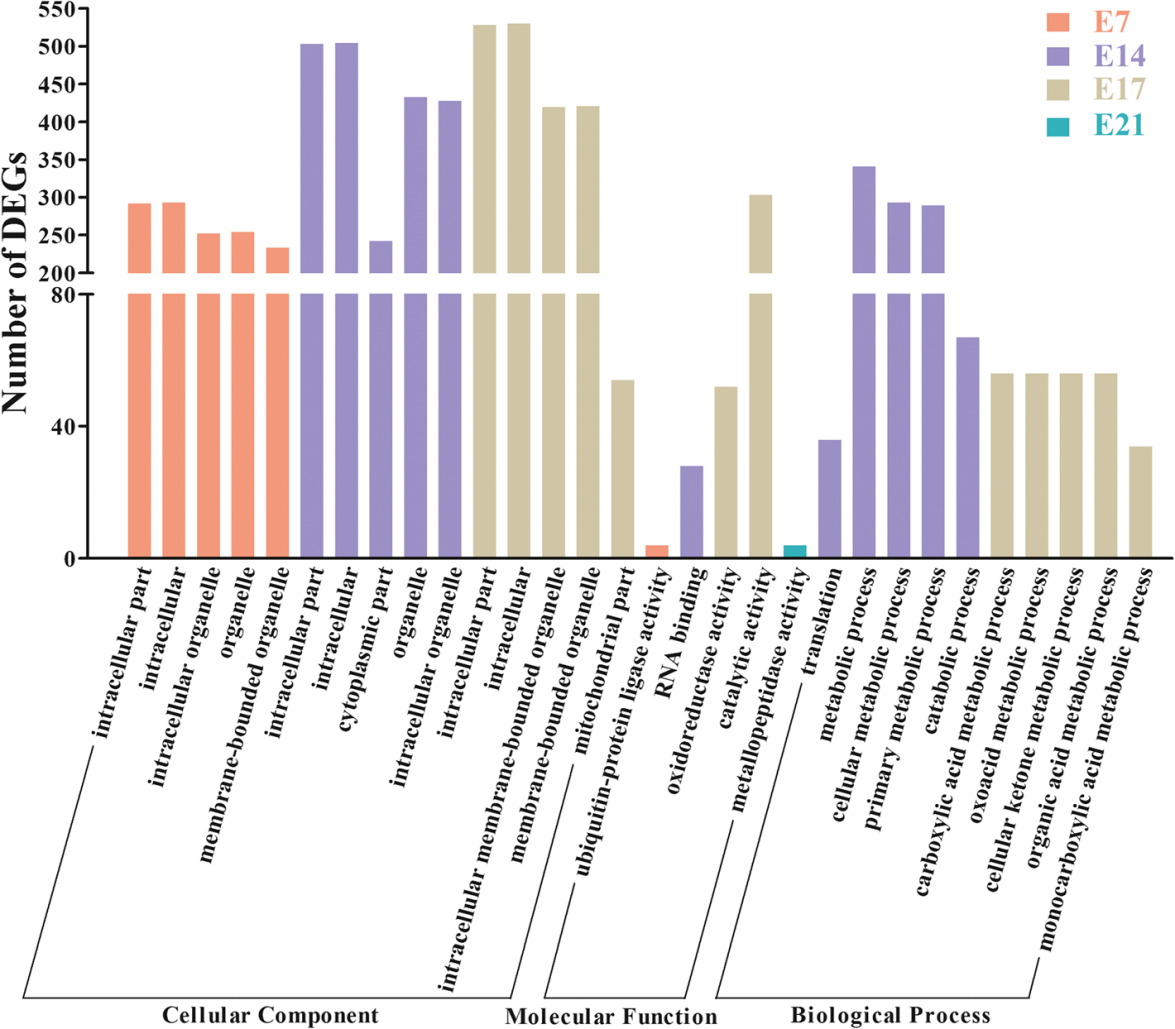
GO analysis of DEGs at different embryonic stages. Significantly enriched GO terms in the biological process, cellular component and molecular function (top five GO terms) categories, respectively (*P* < 0.05). y-axis, the number of DEGs

Gene ontology (GO) and KEGG analysis were performed to analyze biological function of DEGs. DEGs at E7 were enriched in the GO term, the ubiquitin-protein ligase activity. No GO term was found at E12. At E14, the highly enriched GO terms were related to biological processes, such as translation, metabolic process, catabolic process, and RNA binding. At E17, GO terms, such as carboxylic acid metabolic process, oxoacid metabolic process, cellular ketone metabolic process, oxidoreductase activity, and catalytic activity, were enriched. The GO term metallopeptidase activity was enriched for E21 (Fig. 3).

**Fig. 4:**
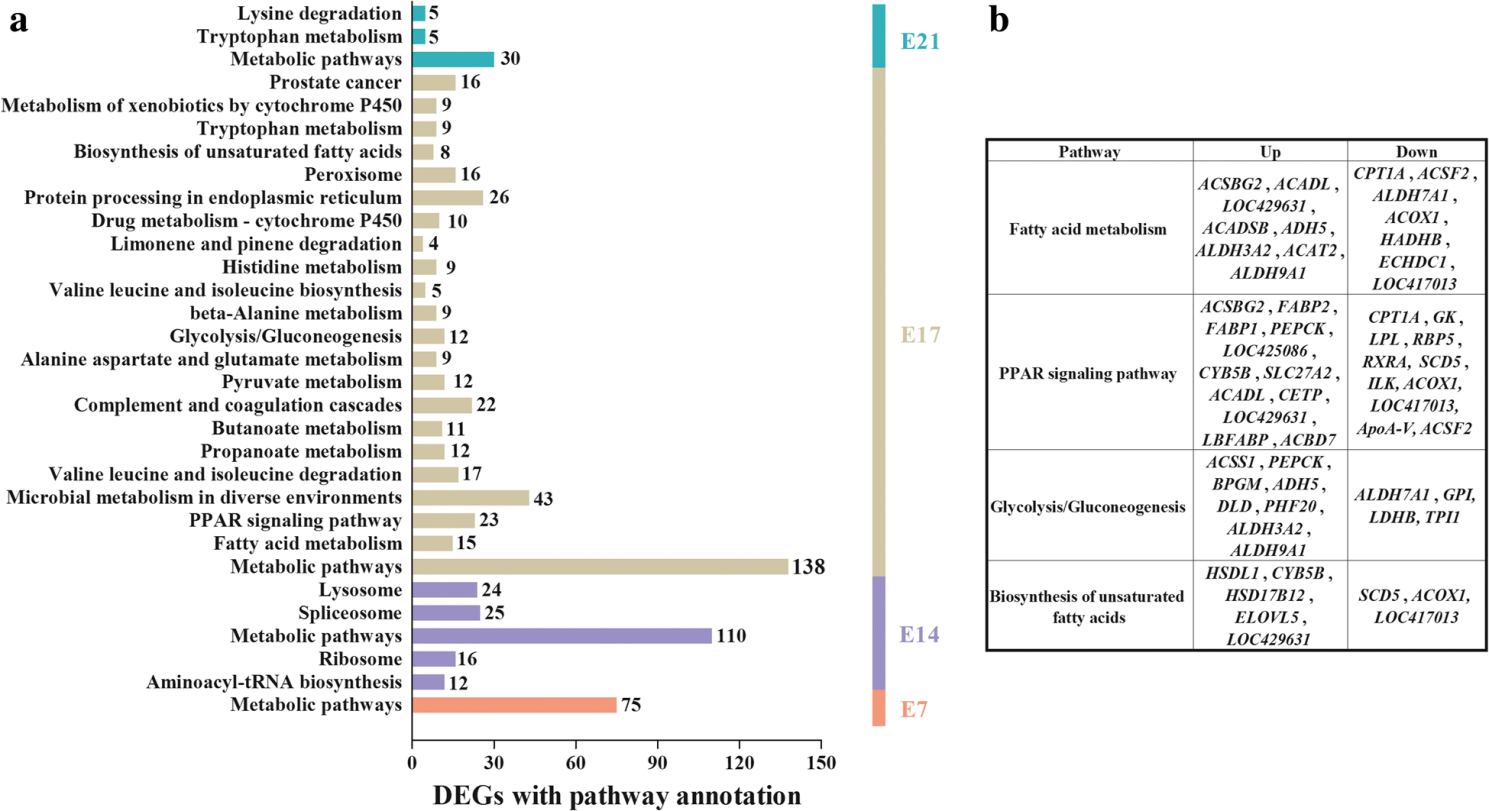
KEGG analysis of DEGs at the five time point. **a** The significantly enriched pathways (Q ≤ 0.05) at five embryonic stages. x-axis, the number of DEGs with pathway annotation; **b** The significantly enriched signaling pathways directly related to lipid metabolism at E17. Up and Down, up- and down-regulated DEGs in the fat line

Similar to GO analysis, no significant signaling pathway was found for E12 in KEGG analysis (Fig. 4a). While for the remaining 4 embryonic stages (E7, E14, E17, and E21), a common signaling pathway, the metabolic pathway, was found. At E14, pathways mainly related to aminoacyl-tRNA biosynthesis, ribosome, spliceosome and lysosome were enriched. A large number of signaling pathways were enriched for DEGs at E17, including those directly related to lipid metabolism, such as fatty acid metabolism and biosynthesis, PPAR signaling pathway, and glycolysis (Fig. 4b). At E21, the amino acid metabolism (tryptophan metabolism and lysine degradation) pathway was enriched.

**Fig. 5:**
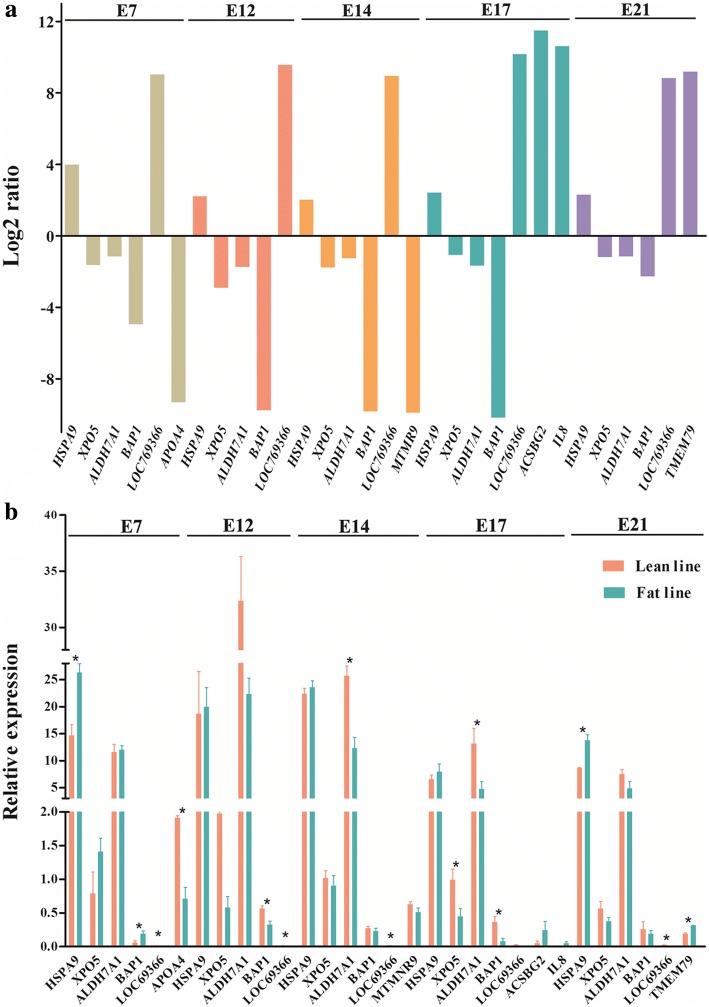
Validation of DEGs by qRT-PCR. **a** 12 DEGs identified by digital gene expression. Y-axis, log2 (fold change of gene expression levels of the fat line vs. the lean line); **b** qRT-PCR results of 12 DEGs in both chicken lines. *, significant or approximately significant (*P* < 0.05)

We validated the digital gene expression results of 12 DEGs (a total of 21 DEGs were selected from 6 common DEGs and top 3 DEGs of each embryonic stage, and 9 of them were duplicated) by qRT-PCR assays. The qRT-PCR results of 2 genes, *LOC77109* and keratin 75 (*KRT75*), were not analyzed because of their low expression levels (Ct > 32). Otherwise, poor reproducibility and unreliable results could be obtained. For the remaining 10 genes, we found out that 4 genes (*APOA4*, *LOC769366*, *HSPA9* and *BAP1*) at E7, 2 genes (*BAP1* and *LOC769366*) at E12, 2 genes (*LOC769366* and *ALDH7A1*) at E14, 4 genes (*LOC769366*, *BAP1*, *XPO5* and *ALDH7A1*) at E17, and 3 genes (*TMEM79*, *LOC769366* and *HSPA9*) at E21, were validated to be significant or approximately significant, respectively (Fig. 5b). Moreover, *APOA4* at E7, *HSPA9* at E7 and E21, *BAP1* at E12 and E17, *ALDH7A1* at E14 and E17, *XPO5* at E17, *TMEM79* at E21 had similar trends for the digital gene expression and qRT-PCR results, whereas *LOC769366* at all five time points and *BAP1* at E7 had opposite trends. In all, our qRT-PCR results were consistent with the digital gene expression results (correlation coefficient *r* = 0.73).

### Comparative proteomics on embryonic hepatic tissues

Quantitative proteomics data were generated from 15 high-quality and reproducible 2-D DIGE maps (Additional file 7). On each gel, about 2000 protein spots were detected. The isoelectric points of most proteins focused on 4 to 10, while molecular weights were distributed from 5 to 100 kDa.

**Fig. 6:**
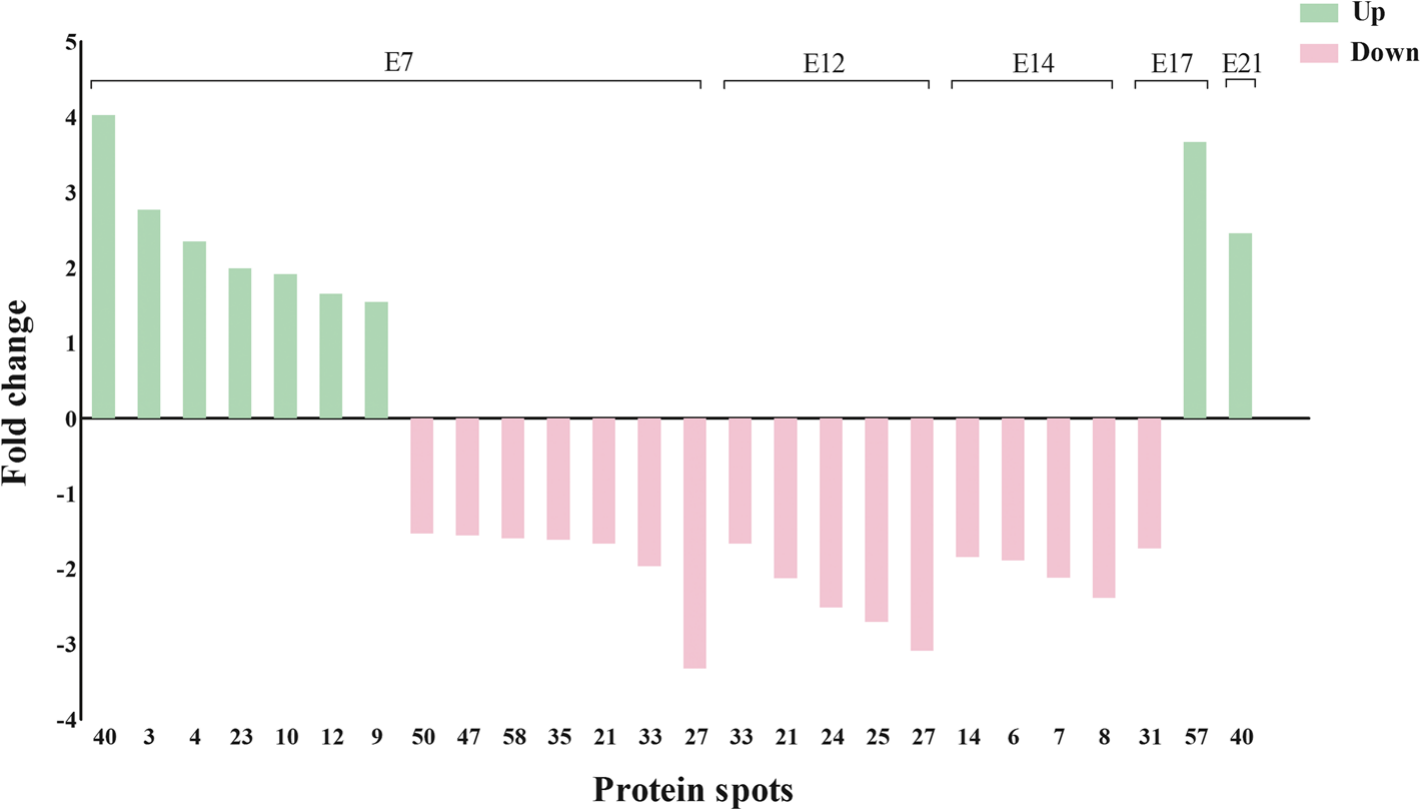
A total of 26 protein spots analyzed between the two lines at five time points. x-axis, differentially expressed protein spots. y-axis, fold change-Ratio (Fat/Lean). + and -, up- and down-regulated in the fat line

**Fig. 7:**
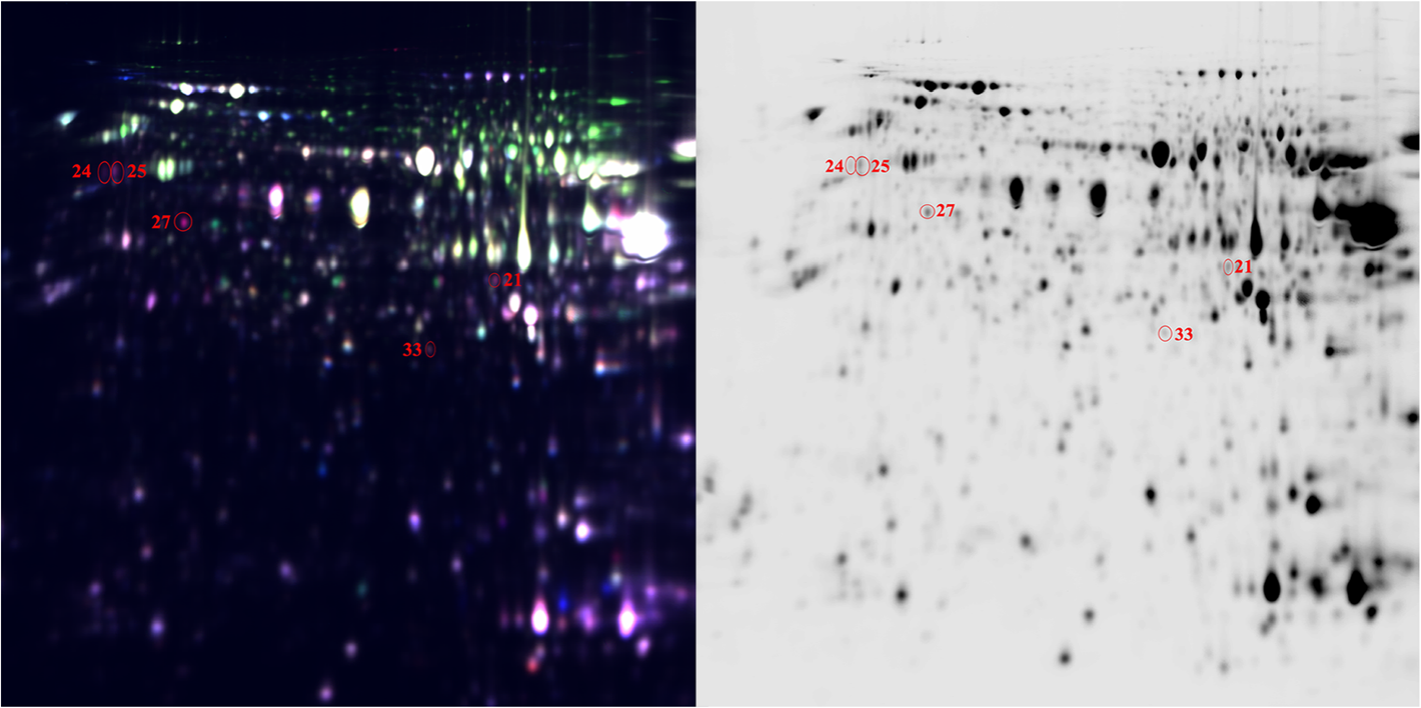
Representative 2D DIGE images. At E12, 5 differentially expressed protein spots were identified

We compared protein profiles between the fat and lean lines at each time point to find key proteins related to lipid metabolism. We found 14 (protein spots 3, 4, 9, 10, 12, 21, 23, 27, 33, 35, 40, 47, 50, 58), 5 (protein spots 21, 24, 25, 27, 33), 4 (protein spots 6, 7, 8, 14), 2 (protein spots 31, 57) and 1 (protein spot 40) differentially expressed protein spots (*P* < 0.05 and fold changes ≥1.5) for the five time points (E7, E12, E14, E17 and E21), respectively (Figs. 6 and 7). Among these differentially expressed protein spots, 3 protein spots (21, 27, 33) were common to E7 and E12, and the protein spot 40 was common to E7 and E21. At E7, differentially expressed protein spots 3, 4, 9, 10, 12, 23, 40 and 21, 27, 33, 35, 47, 50, 58 were up-regulated and down-regulated in the fat line, respectively. At E12 (protein spots 21, 24, 25, 27, 33) (Fig. 7) and E14 (protein spots 6, 7, 8, 14), signal intensities of these differentially expressed protein spots in the lean line was obviously stronger than those in the fat line. At E17, the differentially expressed protein spot 31 was down-regulated, whereas spot 57 was up-regulated in the fat line. At E21, differentially expressed protein spot 40 had higher expression abundance in the fat line.

A total of 26 differentially expressed protein spots were found at the five embryonic stages between the fat and lean lines, and after removing the 4 protein spots that repeated multiple times, 22 differentially expressed protein spots were identified by MALDI-TOF-MS. Except that 2 protein spots (4, 6) could not be identified, the remaining 20 protein spots matched to 17 proteins in the chicken protein database, and to 3 other proteins in protein databases of other species (Table 2).

**Table 2:** Features of the 20 differentially expressed proteins identified by MALDI-TOF-MS

Stage	Protein spot No.	Fold change-ratio (Fat/Lean)	Accession No. (NCBInr/UniProt)	Protein name	Molecular weight (kDa)	pI	Protein score	Protein score C.I.%	Subcellular localization
E7	3	2.77	gi|71895483	lysyl-tRNA synthetase	68,330.4	5.89	271	100	Cytoplasm; Mitochondrion
	4	2.35	Unknown protein						
	9	1.54	Q9YHT1	Succinate dehydrogenase [ubiquinone] flavoprotein subunit, mitochondrial	74,024.6	6.65	63	94.987	Mitochondrion
	10	1.91	Q5ZJ08	Tyrosyl-tRNA synthetase, cytoplasmic	59,703.1	6.24	76	99.743	Cytoplasm
	12^a^	1.65	gi|326927880	PREDICTED: transketolase-like [*Meleagris gallopavo*]	87,487.4	8.17	125	100	
	21^a^	−1.67	gi|326936041	PREDICTED: delta(3,5)-Delta(2,4)-dienoyl-CoA isomerase, mitochondrial-like, partial [Meleagris gallopavo]	16,841.6	6.9	99	99.971	
	23	1.99	gi|57530789	Thioredoxin domain-containing protein 5	47,249.1	5.61	167	100	
	27	−3.33	gi|45384226	Sulfotransferase	36,333.5	5.89	129	100	
	33	−1.97	gi|45384222	Heat shock protein beta-1	21,715	5.77	135	100	Cytoplasm; Nucleus; Mitochondrion
	35^a^	−1.62	gi|109032822	PREDICTED: transcription factor BTF3-like [*Macaca mulatta*]	17,669.1	5.74	181	100	
	40	4.02	O57535	Nucleoside diphosphate kinase	17,447.9	7.72	75	99.661	Cytoplasm; Cell membrane
	47	−1.56	gi|50751047	PREDICTED: inosine triphosphate pyrophosphatase	22,533.4	5.8	142	100	
	50	−1.53	gi|50755667	PREDICTED: NADH dehydrogenase [ubiquinone] 1 beta subcomplex subunit 10	20,769.2	5.98	181	100	
	58	−1.6	O57535	Nucleoside diphosphate kinase	17,447.9	7.72	123	100	Cytoplasm; Cell membrane
E12	21^a^	−2.13	gi|326936041	PREDICTED: delta(3,5)-Delta(2,4)-dienoyl-CoA isomerase, mitochondrial-like, partial [Meleagris gallopavo]	16,841.6	6.9	99	99.971	
	24	−2.52	gi|129293	Ovalbumin	43,195.6	5.19	197	100	Extracellular region
	25	−2.71	gi|129293	Ovalbumin	43,195.6	5.19	370	100	Extracellular region
	27	−3.09	gi|45384226	Sulfotransferase	36,333.5	5.89	129	100	
	33	−1.67	gi|45384222	Heat shock protein beta-1	21,715	5.77	135	100	Cytoplasm; Nucleus; Mitochondrion
E14	6	−1.89	Unknown protein						
	7	−2.12	P84407	Alpha-fetoprotein	72,857.6	6.26	66	97.655	Secreted
	8	−2.39	P84407	Alpha-fetoprotein	72,857.6	6.26	71	99.187	Secreted
	14	−1.85	gi|86129440	Coronin-1C	53,744.3	6,22	109	99.997	Intracellular
E17	31	−1.73	P08250	Apolipoprotein A-I	30,661.1	5.58	72	99.292	Secreted
	57	3.67	gi|56119000	Intestinal fatty acid-binding protein	15,079.7	6.62	247	100	Intracellular part
E21	40	2.46	O57535	Nucleoside diphosphate kinase	17,447.9	7.72	75	99.661	Cytoplasm; Cell membrane

^a^represents proteins matched to other databases. Unknown protein indicates protein spots could not be identified

These 17 proteins matching the chicken protein database included 3 proteins which had been identified twice, the alpha-fetoprotein (protein spots 7 and 8), ovalbumin (OVAL) (protein spots 24 and 25) and nucleoside diphosphate kinase (NDK) (protein spots 40 and 58), possibly due to different protein isoforms or posttranslational modifications (Table 2). For the 14 different DEPs, after GO analysis, 9 of them could be classified into 3 categories with regard to the molecular function: catalytic activity (67%), binding (22%), and transporter activity (11%). However, for the remaining 5, we could not find any hits related to the molcular function. These DEPs were mainly involved in the PPAR signaling pathway, citrate cycle (TCA cycle), fat digestion and absorption, oxidative phosphorylation, aminoacyl-tRNA biosynthesis and MAPK signaling pathways.

### Integrated analysis on transcriptome and proteome data

**Fig. 8:**
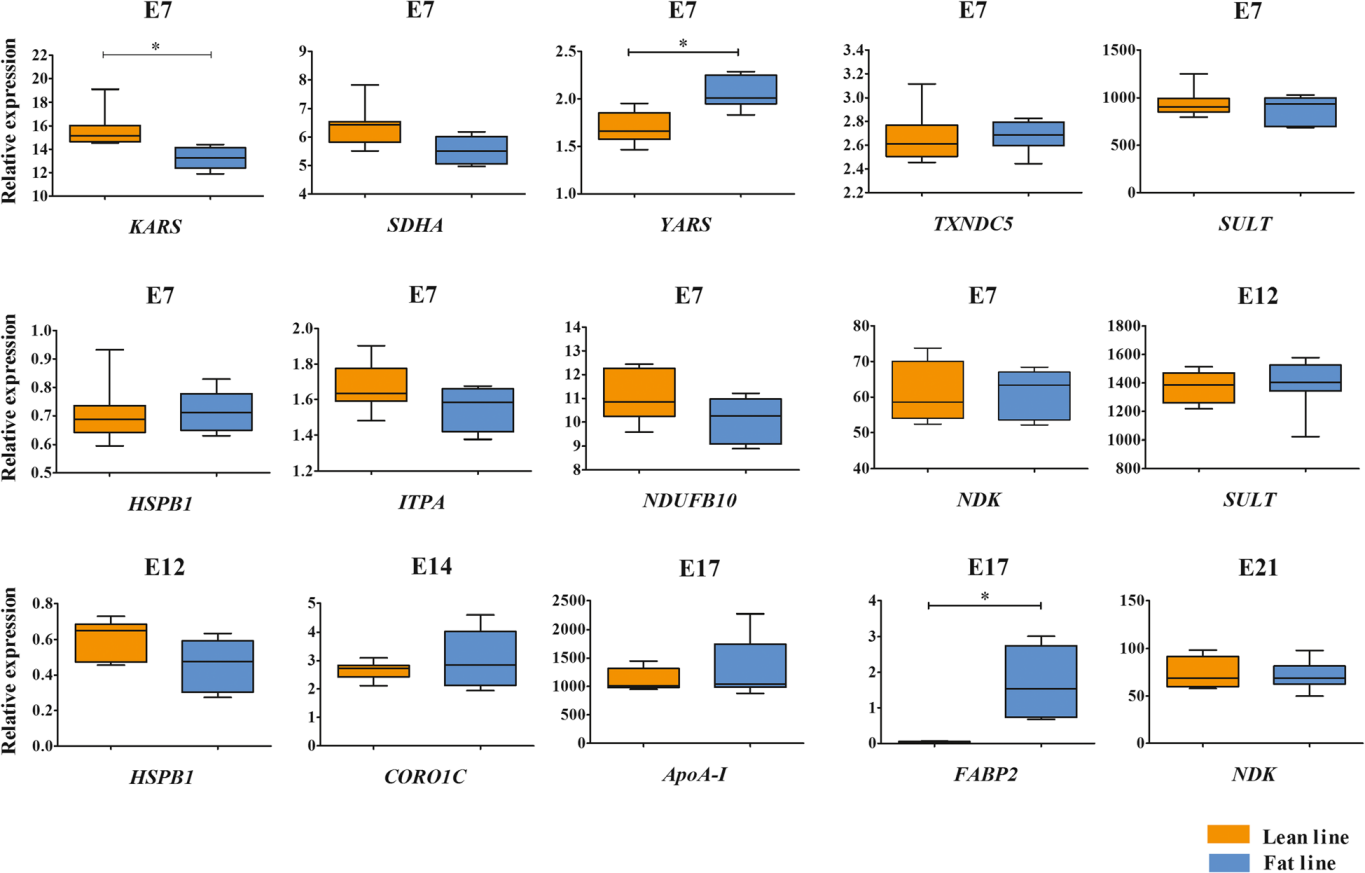
Validation by qRT-PCR of proteins identified by comparative proteomics. After qRT-PCR analyses on 12 genes selected from 20 proteins found by comparative proteomics, consistent results between digital gene expression and qRT-PCR were found. *KARS, YARS* and *FABP2* were significantly differentially expressed (*, *P* < 0.05)

For the 14 DEPs identified by the comparative proteomics, 2 of them, alpha-fetoprotein and OVAL, had no data in the digital gene expression experiment. The transcriptional abundances of the remaining 12 DEPs were validated by qRT-PCR, at the same time points when significant differences of protein abundances were found. However, only were three genes, lysyl-tRNA synthetase (*KARS*), tyrosyl-tRNA synthetase (*YARS*) and intestinal fatty acid-binding protein (*FABP2*), validated to be significantly differentially expressed at E7 and E17, respectively (Fig. 8). When comparing the digital gene expression and qRT-PCR results, *KARS* and *YARS* had opposite expression trends at E7, and *FABP2* had a similar expression trend at E17. In addition, similar expression patterns to their digital gene expression results were obtained for 5 genes, succinate dehydrogenase [ubiquinone] flavoprotein subunit (*SDHA*) at E7, thioredoxin domain-containing protein 5 (*TXNDC5*) at E7, sulfotransferase (*SULT*) at E7 and E12, inosine triphosphatase (*ITPA*) at E7, and *ApoA-I* at E17. By contrast, opposite expression levels were found for 4 other genes, NADH dehydrogenase [ubiquinone] 1 beta subcomplex subunit 10 (*NDUFB10*) at E7, heat shock protein beta-1 (*HSPB1*) at E7 and E12, coronin-1C (*CORO1C*) at E14, and *NDK* at E7 and E21. Furthermore, a general correlation analyses showed that digital gene expression and qRT-PCR results were consistent with each other (correlation coefficients *r* = 0.86 and 0.84 for the lean and fat lines, respectively).

**Fig. 9:**
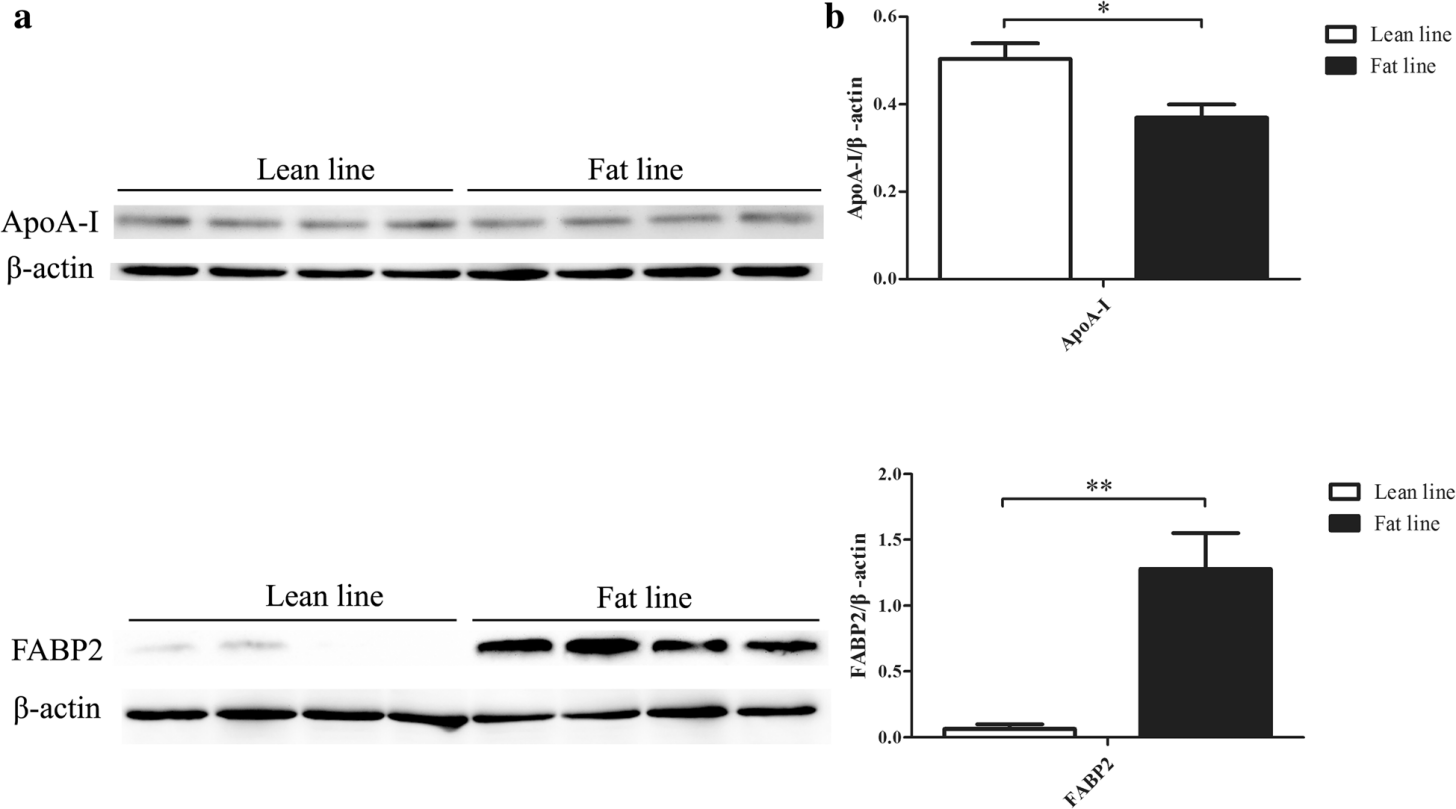
Western blots for ApoA-I and FABP2. Four hepatic tissue samples at E17 were assayed for the lean and fat chicken lines, respectively. For ApoA-I, the lean line were significantly higher than the fat line (a, b). In contrast, for FABP2, the fat line had strikingly higher protein level than the lean line (a, b)

To validate the proteomics results, the western blot experiment was performed for ApoA-I and FABP2. We selected these two proteins, based on the facts that ApoA-I antibody is prepared in the lab and ready-to-use, FABP2 antibody is commercially available and the antigenic epitope of the commercial FABP2 antibody is relatively conserved (72% similarity to human). Western blot results confirmed that ApoA-I and FABP2 had significantly differential abundance at E17 (Fig. 9a, b).

Furthermore, joint analysis on mRNA and protein expression data based on qRT-PCR results and proteomics results was performed, to see if the mRNA and protein abundance levels of 12 DEPs were consistent with each other. The 12 DEPs were classified into two main groups. Group I contained 3 proteins significantly differentially expressed at both transcriptional and protein expression levels, which could be divided into two subgroups. Subgroup 1 included 2 proteins, in which YARS and FABP2 were significantly higher in the fat birds at E7 and E17. In contrast, in subgroup 2, the protein level of KARS was opposite to its transcriptional level at E7. In Group II, 9 DEPs were not significantly differentially expressed at the transcriptional levels, which could also be classified into 2 subgroups. Subgroup 1 has 6 DEPs with well-matched tendency of transcriptional levels, including TXNDC5, SULT, ITPA, NDUFB10, NDK (protein spot 58) at E7, HSPB1 at E12. Subgroup 2 has 6 DEPs with opposite transcriptional trends, including SDHA, HSPB1 at E7, SULT at E12, CORO1C at E14, ApoA-I at E17, NDK (protein spot 40) at E7 at E21. Subgroups 1 and 2 shared three genes, SULT, HSPB1 and NDK, showing different patterns of transcriptional abundances at different embryonic stages (Table 3).

**Table 3:** Joint analyses on differentially expressed genes and proteins in qRT-PCR and comparative proteomics

Group	Stage	Protein name	Gene name	DEPs in comparative proteomics	Differential expression genes in qRT-PCR	Molecular function	Biological process
Group I-subgroup 1	E7	Tyrosyl-tRNA synthetase, cytoplasmic	*YARS*	Y (Fat > Lean)	Y (Fat > Lean)	Aminoacyl-tRNA ligase activity	Amino acid synthesis
	E17	Intestinal fatty acid-binding protein	*FABP2*	Y (Fat > Lean)	Y (Fat > Lean)	Lipid binding, transporter activity	Lipid transport
Group I-subgroup 2	E7	lysyl-tRNA synthetase	*KARS*	Y (Fat > Lean)	Y (Lean > Fat)	Aminoacyl-tRNA ligase activity	Amino acid synthesis
Group II-subgroup 1	E7	Thioredoxin domain-containing protein 5	*TXNDC5*	Y (Fat > Lean)	N (Fat > Lean)	Disulfide oxidoreductase activity	Protein folding
	E7	Sulfotransferase	*SULT*	Y (Lean > Fat)	N (Lean > Fat)	Sulfotransferase activity, transferase activity	Sulfuryl transfer
	E7	PREDICTED: inosine triphosphate pyrophosphatase	*ITPA*	Y (Lean > Fat)	N (Lean > Fat)	Pyrophosphatase activity	Nucleotide metabolic process
	E7	PREDICTED: NADH dehydrogenase [ubiquinone] 1 beta subcomplex subunit 10	*NDUFB10*	Y (Lean > Fat)	N (Lean > Fat)	Binding, NADH dehydrogenase (quinone) activity	Energy metabolism
	E7	Nucleoside diphosphate kinase (protein spot 58)	*NDK*	Y (Lean > Fat)	N (Lean > Fat)	Nucleoside diphosphate kinase activity	Lipid transport
	E12	Heat shock protein beta-1	*HSPB1*	Y (Lean > Fat)	N (Lean > Fat)	Molecular chaperone-mediated protein folding	Lipid clearance
Group II-subgroup 2	E7	Nucleoside diphosphate kinase (protein spot 40)	*NDK*	Y (Fat > Lean)	N (Lean > Fat)	Nucleoside diphosphate kinase activity	Lipid transport
	E7	Succinate dehydrogenase [ubiquinone] flavoprotein subunit, mitochondrial	*SDHA*	Y (Fat > Lean)	N (Lean > Fat)	Adenyl nucleotide binding, succinate dehydrogenase activity	Energy metabolism
	E7	Heat shock protein beta-1	*HSPB1*	Y (Lean > Fat)	N (Fat > Lean)	Molecular chaperone-mediated protein folding	Lipid clearance
	E12	Sulfotransferase	*SULT*	Y (Lean > Fat)	N (Fat > Lean)	Sulfotransferase activity, transferase activity	Sulfuryl transfer
	E14	Coronin-1C	*CORO1C*	Y (Lean > Fat)	N (Fat> Lean)	Cytoskeletal protein binding	Actin cytoskeleton organization
	E17	Apolipoprotein A-I	*ApoA-I*	Y (Lean > Fat)	N (Fat > Lean)	Cholesterol binding, cholesterol transporter activity	Lipid transport
	E21	Nucleoside diphosphate kinase (protein spot 40)	*NDK*	Y (Fat > Lean)	N (Lean > Fat)	Nucleoside diphosphate kinase activity	Lipid transport

Y and N represent yes and no, respectively

## Discussion

Nowadays, to reduce fat deposition is still an important goal for commercial chicken breeding program [5]. In avian species, the liver is the main site of de novo fatty acid synthesis [7], while the adipose tissue serves mainly as a storage tissue [6, 15]. Accumulation of triacylglycerols in adipose tissue is directly related with hepatic lipogenesis [9]. Using NEAUHLF, a suitable animal model to study the molecular mechanism of adipose tissue growth and development, we previously found genes and molecular pathways important for hepatic lipid metabolism (*PPARγ*, *LBFABP*, ApoA-I, AFABP and glycol-metabolism) in the liver and adipose tissues at 1, 4 and 7 weeks of age by microarray and proteomics analyses [10–13]. However, whether there are differences on hepatic lipid metabolism between the two lines in embryonic stages remains unknown. In the current study, we compared transcriptome and proteome profiling on hepatic tissues sampled from 5 different embryonic stages between the two lines. Integrated mRNA and protein expression data showed that 8 DEPs (2 and 6 in subgroups 1 of Group I and II, respectively), YARS, TXNDC5, SULT, ITPA, NDUFB10, NDK (protein spot 58) at E7, HSPB1 at E12, and FABP2 at E17, had similar trend of transcriptional levels. However, 7 DEPs (1 and 6 in subgroups 2 of Group I and Group II, respectively), KARS, SDHA, HSPB1 at E7, SULT at E12, CORO1C at E14, ApoA-I at E17, and NDK (protein spot 40) at E7 at E21, had opposite transcriptional trends. Inconsistency between mRNA and protein expression levels probably comes from a variety of factors involved in the regulation of mRNA and protein abundances, such as (post-) transcriptional and (post-) translational regulation, protein modification, protein-protein interaction, and other regulatory mechanisms as well [16]. Among these 12 DEPs, FABP2, NDK and ApoA-I were involved in lipid transport; HSPB1 was related to lipid clearance; SULT and TXNDC5 could participate in hepatic lipid metabolism through PPARγ and apolipoprotein B (ApoB); SDHA and NDUFB10 were involved in energy metabolism. However, for KARS, YARS, ITPA and CORO1C, no reports on their direct relationship with lipid metabolism are available.

Proteins related to lipid transport, FABP2, NDK and ApoA-I, were found to be differentially expressed between the two lines in the present study. FABP2, also known as intestinal FABP (I-FABP), had higher protein and mRNA abundances in the fat broilers at stages of rapid growth and development (E12, E14, E17 and E21) found by both transcriptome and proteome analyses, which were also validated to be significantly differentially expressed between the two lines at E17 by western blot and qRT-PCR. FABP2 is involved in lipid metabolism, especially in the uptake, intracellular metabolism and transport of long chain fatty acids [17, 18]. FABP2 may also influence mitochondrial fatty acid oxidation and free cholesterol transport by regulating gene expression and interaction with nuclear receptors [19]. The higher expression level of FABP2 in the fat birds may imply that the fat birds have stronger capacity of lipid transport.

In the present study, mass spectrometry results showed NDK had two isoforms (protein spots 40 and 58). The abundance of protein spot 40 in the fat line was significantly higher than that in the lean line at E7 and E21, and the transcriptional level of NDK was higher in the lean line compared to the fat line at E7 and E21. However, for protein spot 58 at E7, its mRNA and protein abundance was down-regulated in the fat birds. Human studies also indicate that NDK have two isoforms. Though the two isoforms are closely related in amino acid sequences (88% identity), they display significant differences in cellular functions [20]. NDK catalyzes phosphoryl transfer from a nucleoside triphosphate to a nucleoside diphosphate, and functions in the metabolic pathway [21]. NDK regulates synaptic vesicle internalization, where the dynamin GTPase is required to function [22], and is indispensable in energy metabolism in development [23]. Recently, it was found that NDK is a lipid-dependent mitochondrial switch in both phosphor-transfer and inter-membrane cardiolipin transfer, which relates to apoptotic signaling and other putative functions, potentially important in lipid metabolism [24, 25].

In addition, for ApoA-I that is also involved in lipid transport, we found that the lean birds had a significantly higher ApoA-I protein abundance at E17. However, no transcriptional difference for *ApoA-I* in the embryonic liver was found between the fat and lean broilers at E17, suggesting the *ApoA-I* may be regulated at the translational level. We previously examined ApoA-I and its association with fat deposition using genetics, gene expression and proteomics methods in adipose tissues [12, 13]. ApoA-I is a major component of high-density lipoprotein (HDL) in the plasma [26], and can promote cholesterol efflux from peripheral tissues to the liver to keep body cholesterol in balance [27]. ApoA-I can not only function as a key lipoprotein to transport cholesterol, but can also inhibit fatty acid synthesis in mice [28]. Thus, we speculate that ApoA-I can influence embryonic liver lipid metabolism, and contribute to the striking differences of abdominal fat deposition between the fat and lean chicken lines.

HSPB1 could be related to lipid clearance, and protein levels were significantly higher in the lean birds at E7 and E12 as revealed by quantitative proteomics. At E12, both transcriptional and protein expression levels of HSPB1 agreed with each other. But at E7, the protein level of HSPB1 was opposite to its transcriptional level, which may be due to post-translational modification [29]. HSPB1 (also known as HSP27) serves as an ATP-independent chaperone [30]. In mammals, it has been reported that obese subjects had higher anti-HSP27 antibody levels [31], and induction of HSP27 may blunt the adverse effect of fat overexposure on insulin function [32]. In diabetic hearts, the phosphorylation of HSP27 enhanced lipoprotein lipase activity and promoted hydrolysis of triglyceride-rich lipoproteins to fatty acids [33]. Phosphorylated HSP27 promotes autophagy and hepatic lipid clearance via autophagy-lysosome pathway in human hepatic cells [34]. We previously found that HSP27 protein was down-regulated in the abdominal adipose tissue of fat birds [12, 13], and here we found that HSP27 was down-regulated in the liver tissue of fat birds at E7 and E12. Taken together, our data strongly suggest that HSP27 might be important for hepatic lipid metabolism in chickens.

We identified also 2 other proteins, SULT and TXNDC5, potentially important for lipid metabolism in chicken embryos. The protein and transcriptional abundance of SULT was all higher in the lean line at E7, whereas the protein level of SULT was opposite to its transcriptional level at E12. In humans, SULT shows nuclear translocation and can be post-translationally modified [35]. The different expression patterns of SULT in protein and transcription levels could also be due to post-translational modification at E12 [35]. Sulfotransferase is a transferase enzyme known to catalyze the transfer of a sulfo group from a donor molecule to an acceptor group of numerous substrates [36], and reactive groups for a sulfonation via sulfotransferases may be part of a protein, lipid, or steroid [37]. It is capable of responding to inflammatory cues and controlling lipid metabolism by PPARγ in humans [38]. The different expression of SULT between the two lines at E7 and E12 showed that SULT may participate in hepatic lipid metabolism via PPARγ in the chicken. Both transcriptional and protein expression levels of TXNDC5 was higher in the fat chickens at E7. TXNDC5 has a protein disulphide isomerase-like domain, and belongs to the thioredoxin family, which is thought to catalyze disulphide formation to aid protein folding or to regulate protein function against endoplasmic reticulum stress induced by oxidative insults. TXNDC5 protein and mRNA levels were significantly associated with hepatic fat content in *ApoE*-knockout mice [39]. TXNDC5 modulated adiponectin signalling by interacting with adiponectin receptor 1 (AdipoR1) [40] and contributed to increased risk of hepatocellular carcinoma development [41]. Moreover, we found also that proteins in the oxidative stress pathway were differentially expressed. Oxidative stress can alter the expression of *ApoB*, and VLDL secretion [42]. Therefore, TXNDC5 could couple with the control of *ApoB* levels by the oxidative stress pathway, to exert its effect on subsequent hepatic lipid metabolism.

Succinate dehydrogenase (SDH) and NDUFB10 could participate in energy metabolism. The protein abundance of SDHA was higher in the fat line, whereas the protein level was opposite to its transcriptional level at E7. The mRNA and protein levels of NDUFB10 were higher in the lean lines at E7. SDH is known as succinate-ubiquinone oxidoreductase, a complex of the mitochondrial respiratory chain. The complex is composed of four nuclear-encoded subunits (SDHA, SDHB, SDHC and SDHD). It has a role in citric acid cycle and mitochondrial energy generation in mammals [43]. NDUFB10 is a subunit of the NADH dehydrogenase (ubiquinone) complex, located in the mitochondrial inner membrane [44]. The different expression of SDHA and NDUFB10 indicated that the fat birds and lean birds had differences in regard to energy metabolism.

We found two Aminoacyl-tRNA synthetases (KARS and YARS), which are important for amino acid synthesis, were differentially expressed between the fat and lean birds in liver tissue at E7. Both the protein and mRNA expression levels of YARS were significantly higher in the fat birds at E7. The protein level of KARS was significantly higher in the fat birds at E7 and the mRNA level was significantly higher in the lean birds at E7. They catalyze the aminoacylation of tRNA by their cognate amino acid [45]. A number of studies reported that the functions of ARSs were also associated with RNA splicing, immune responses, angiogenesis and cell fate determination besides protein synthesis [46]. But the functions of the two proteins in chicken hepatic lipid metabolism are not very clear.

Two proteins (ITPA, CORO1C) were found to be differentially expressed between the two lines at E17 and E14, respectively. ITPA had higher protein and mRNA abundances in the lean broilers at E7. The protein abundance of CORO1C was significantly higher in the lean broilers at E14, whereas transcriptional abundance was higher in the fat broilers. However, their functions on lipid metabolism remain to be investigated.

In poultry, fat deposition depends on the availability of plasma triglycerides, which are transported as components of lipoproteins [9]. Fattening therefore is in connection with three aspects of lipid metabolism: (1) lipid synthesis; (2) lipid transport; and (3) lipid utilization. It is reported previously that the liver of the avian embryo has the capacity for lipoprotein synthesis, secretion and β-oxidation [47], though lipogenesis within the embryonic liver tissue is low [48]. Moreover, the chick embryo liver has a very high capacity for β-oxidation, and fatty acid oxidation provides most of the energy that is required for embryo development [49]. As mentioned above, we found that genes/proteins related to lipid transport and energy metabolism were differentially expressed in the embryonic liver between the fat and lean lines. As a result, differences of transport and utilization of lipids as well, will appear. This could be one of the underlying reasons for the significant difference of fat deposition between our two chicken lines, starting at 7 days posthatch [50].

## Conclusions

Molecular differences related to lipid transport, lipid clearance and energy metabolism exist for hepatic lipid metabolism at embryonic stages between the fat and lean chicken lines, which might contribute to the striking differences of abdominal fat deposition at post-hatch stages.

## Methods

### Chicken embryos

The fertilized chicken eggs were chosen from the 14th generation of Northeast Agricultural University broiler lines divergently selected for high and low abdominal fat content (NEAUHLF), 200 each for the fat and lean lines, respectively. The fertilized chicken eggs were all hatched in the same conditions at Northeast Agricultural University hatchery.

### Collection of liver tissues

Liver tissues were collected from chicken embryos at the five embryonic stages, E7, E12, E14, E17 and E21. The start of the incubation period was referred to as “E1” (1-day-old embryos) and “E21”, for newly hatched birds. Liver tissues were collected from chicken embryos under aseptic conditions, frozen immediately in liquid nitrogen, and stored at − 80 °C.

### RNA sample preparation and digital gene expression

Except 30 liver samples from embryos at E7 were pooled together for RNA extraction, for the remaining four embryonic stages, 15 samples were used, respectively. Total RNAs from the 10 samples were extracted using Trizol (Invitrogen), and RNA quality and concentration were evaluated, to ensure RNA integrity number (RIN) ≥ 9.

Sequencing libraries for digital gene expression analyses were prepared according to the following procedures. Messenger RNAs were purified from 6 μg total RNA, and reversely transcribed into cDNA, which was then digested using the restriction enzyme *Nla*III (recognition sites: 5′-…CATG/GTAC…-3′). Adapter 1 (Illumina) was then ligated to the 5′-end, and the restriction enzyme *Mme*I was then added, which can recognize the nucleotide sequences composed of adapter 1 and CATG, and then cut at the downstream 17 bp site, to produce tags labelled with adapter 1. Then adapter 2 (Illumina) was ligated with these tags, and 15 cycles of PCR were performed. PCR products were run on 6% TBE PAGE and purified, which were then submitted to Illumina HiSeq™ 2000 for digital gene expression analysis (BGI, Shengzhen).

### Bioinformatics analyses of sequencing data

Procedures for analyzing the digital gene expression profiling data were briefly described as follows. Short reads generated were assessed first for their sequence quality and reads of poor quality were discarded. Libraries containing all possible indices were built. Assessment of sequencing quality was performed, such as distribution of distinct tags (Additional file 8), and proportion of clean tags (all > 96%) (Additional file 9). Reference tag library was created by analyzing mRNAs containing the restriction enzyme *Nla*III recognition sites (CATG) and 17 bases of the reference gene sequences, which was then used for the alignment of generated short reads, with only one base mismatch allowed. Unambiguous tags mapped to one gene were annotated and novel transcripts were discovered by comparing to known transcripts in the database. Number of genes and its relationship with the sequencing volume was evaluated (2 M required to identify maximum number of genes, and all 10 libraries > 3 M) (Additional file 10). Annotated genes and their expression levels were determined and used for downstream differential expression and gene pathway analyses. All genes expressed at a level centred around 10 TPM (transcripts per million clean tags). Furthermore, antisense transcripts were also found, but without significant differences in numbers between lines (Additional file 11). Differentially expressed genes were identified according to the methods described previously [51], and false discovery rate was used for multiple testing correction. We used a criteria of FDR ≤ 0.001 and fold changes ≥2. GO and pathway enrichment analysis was performed by using the GO database (http://www.geneontology.org/) [52] and the KEGG pathway (http://www.genome.jp/kegg/) [53].

### Protein sample preparation and 2-D DIGE

There were three replicates for samples at each stage, and each replicate contained a mixture of liver tissues from more than five different animals, respectively. Total proteins were extracted from liver tissues using Trizol (Invitrogen, Carlsbad, CA) according to the manufacturer’s protocol with minor modifications as follows. The liver tissue samples were dissolved in lysis buffer containing 7 M urea, 2 M thiourea, 4% CHAPS, 10 mM Tris (pH 8.5) and 1 × protease inhibitor cocktails (Roche Diagnostics GmbH, Mannheim, Germany). Then, to remove insoluble materials, samples were centrifuged at 25,000 g for 30 min. Total proteins were purified by 2D Clean-Up Kit (GE Healthcare, Chalfont St Giles, UK), and protein concentration was determined by 2D Quant Kit (Amersham Biosciences Corp., Piscataway, NJ). Protein stock solutions were kept at a final concentration of 5 mg/mL. We compared and analyzed 30 samples on a total of 15 gels (two samples on one gel). Details of the experimental design were briefly described as follows. Protein samples labeled with Cy3 or Cy5 fluorescent dyes were loaded on the same gel, together with an internal standard labeled with Cy2. The labeling reaction was carried out using 400 pmol dyes for 50 μg protein, according to the manufacturer’s instructions (GE Healthcare). Each sample was labeled three times to minimize the influence of dye and systematic errors. Then, samples labeled with three different dyes were mixed, and an equal volume of 2 × sample buffer (7 M urea, 2 M thiourea, 4% CHAPS, 50 mM DTT, 2% pharmalytes 3–10 NL) were added. Final sample volume was brought to 350 μL, with additional sample dissolved in a rehydration buffer (7 M urea, 2 M thiourea, 2% CHAPS, 2% pharmalytes 3–10 NL, 20 mM DTT). First-dimension electrophoresis was conducted with the IPGphor3 isoelectric focusing system (GE Healthcare) using IPG strips (18 cm, pH 3–10 NL), with a total focusing time of 8 kVh at 20 °C. Prior to SDS-PAGE, each strip was equilibrated with 15 mL equilibration buffer A (6 M urea, 50 mM Tris-HCl, pH 8.8, 30% glycerol, 2% SDS, 0.002% Bromophenol blue, 10 mM DTT) on a rocking table for 15 min, followed by a treatment of another 15 min in 15 mL equilibration buffer B (6 M urea, 50 mM Tris-HCl, pH 8.8, 30% glycerol, 2% SDS, 0.002% bromophenol blue, 25 mg/mL iodoacetamide). The strips were then loaded onto the 12.5% acrylamide gels, and gels were run under a constant power at 12 °C first with 2 W/strip for 60 min, and then 15 W/strip, until the bromophenol blue reached the bottoms of the gels.

### Scanning and image analysis

Gels were scanned by the Typhoon 9400 scanner (GE Healthcare). Cy2, Cy3, and Cy5 images for each gel were taken at 488/520, 532/580 and 633/670 nm excitation/emission wavelengths, respectively, adjusting the pixel resolution to 100 mm. All gels were scanned at 50 nm resolution, and the intensity was adjusted to ensure that the maximum volume of each image was within 50,000–80,000. Images were cropped to remove areas extraneous to the gel image, using Image Quant V 5.2 (Amersham Biosciences, UK). Image analysis was performed with DeCyder 6.5 (GE Healthcare). The DeCyder BVA module was used to performing comparative cross-gel statistical analysis of all spots, permitting the detection of differentially expressed spots between experimental groups (t-test, *P* < 0.05). Protein spots with a fold change of at least 1.5 were analyzed.

### Spot picking and in-gel digestion

Gels were fixed and stained with Coomassie brilliant blue (CBB). Proteins of interests, as defined by the 2D-DIGE/DeCyder analysis, were excised from the CBB-stained gels. Gel pieces were added into 100 mmol/L NH_4_HCO_3_ solution buffer in 30% acetonitrile, to decolor for 15 min. The precipitate was collected and washed in 100% acetonitrile and put aside at room temperature for 5 min. After vacuum drying, the gel pieces were incubated with modified trypsin (sequencing grade) at a final concentration of 50 ng/uL at 4 °C for 60 min, and then treated in 50 mmol/L NH_4_HCO_3_ for 16 h at 37 °C. Digested peptide mixtures were extracted twice with 0.1% TFA in 60% acetonitrile. Then, the extracted solutions were blended, lyophilized and kept at − 20 °C for further identification by MS.

### MALDI-TOF-TOF MS analysis and database search

For MALDI-TOF-TOF MS analysis, 1 uL of sample was mixed with 1 uL of matrix and loaded onto the MALDI-TOF slides, and the spot number and sample name were recorded. MALDI-TOF-TOF MS analysis was performed on a 4800 Plus MALDI-TOF-TOF™ analyzer (Applied Biosystems, Foster City, CA, USA). The obtained spectra of proteins were submitted for online database searching against NCBInr and Swiss-Prot databases, using MASCOT program (http://www.matrixscience.com). The following parameters were adopted when searching protein databases: 0.1 Da mass tolerance for peptides and 0.3 Da mass tolerance of TOF-TOF fragments, allowed 50 ppm mass outlier error, reduced Min S/N to 20. Only significant hits were accepted, as defined by the MASCOT probability analysis (*P* < 0.05). Positive hits with either protein score confidence interval (CI) % or Ion CI% greater than 95 were considered significant. GO analysis was performed by using the GO database (http://www.geneontology.org/) [52].

### qRT-PCR

Liver samples were prepared as aforementioned. For E7, at least 15 liver samples were combined into one biological replicate. For E12 and E14, 5 liver samples were mixed together. For E17 and E21, only were 3 liver samples grouped together. Three biological replicates were assayed for the validation of 12 DEGs, while three (E7 and E12), four (E14 and E17), and five (E21) biological replicates were assayed for 12 DEPs, respectively. Primers were designed to span introns and the sequences were listed as in Additional file 12.

Total RNA of liver tissues (50-100 mg) was isolated using Trizol (Invitrogen, Carlsbad, California) according to the manufacturer’s recommendations. RNA concentration was determined by spectrophotometry, and RNA quality was checked on 1.5% agarose gel. cDNAs were reverse transcribed from 1 μg of total RNA at 25 °C for 5 min, 42 °C for 60 min, and 70 °C for 15 min, using 0.5 μL an oligo(dT) primer (Takara, Daliang, China), and 1.0 μL ImProm-II reverse transcriptase (Promega, Madison, WI) in a final volume of 20 μL. TATA box-binding protein (*TBP*) was used as an internal reference to normalize the expression data.

qRT-PCR reactions were performed using FastStart Universal SYBR Green Master kit (Roche) on QuantStudio™ Real-time PCR System following cycling conditions: 1 cycle at 50 °C for 2 min and 95 °C for 10 min, 40 cycles at 95 °C for 15 s and 60 °C for 1 min, and a melting curve analysis (95 °C for 15 s, 60 °C for 1 min and 95 °C for 30 s). Relative expression levels were calculated using the 2^−ΔCt^ method [54]. Data were analyzed using the T-test and *p*-value <0.05 was considered to be significant.

### Western blot

Liver samples used in qRT-PCR at E17 were homogenized in Radio Immunoprecipitation Assay (RIPA) buffer (50 mM Tris, PH 7.4, 150 mM NaCl, 1% Triton X-100, 1% sodium deoxycholate, 0.1% SDS) with 10 mM of a protease inhibitor Phenylmethanesulfonyl fluoride, and centrifuged at 13,000×*g* for 10 min. The total protein samples were separated by 12% SDS-PAGE and transferred onto nitrocellulose membranes. After blocking with 5% nonfat milk in PBS with Tween-20 (PBST) for 2 h at room temperature, the membranes were incubated with antibodies against ApoA-I (1:800 dilution; Abmart, Shanghai, China), FABP2 (1:800 dilution; Santa Cruz, Dallas, USA) or β-actin (1:1000 dilution; TransGen, Beijing, China) overnight at 4 °C. The rabbit anti-chicken ApoA-I antibody was prepared in our lab. Then, the membranes were washed with PBST for five times and incubated with goat anti-rabbit or goat anti-mouse conjugated with horseradish peroxide (1:5000 dilution; Beyotime, Beijing, China) for 1 h at room temperature. Following five washes with PBST, signals were tested using super ECL kit (HaiGene, Harbin, China). Densitometric measurement of the bands was analyzed using a laboratory imaging and analysis system (lane 1D), and then t-test was used for the statistical significance analysis.

## Additional files

Additional file 1: **Figure S1.** Number of genes expressed and novel transcripts. L and F represent the lean and fat chicken lines, respectively. (DOC 177 kb)

Additional file 2: **Table S1.** DEGs identified in liver of E7 embryos between the fat and lean lines. RawIntensity, raw copy number of tag; TPM, normalized expression of tag; log2 Ratio, log2 (fold changes of differentially expressed genes); FDR, false-discovery rate. (XLSX 72 kb)

Additional file 3: **Table S2.** DEGs identified in liver of E12 embryos between the fat and lean lines. (XLSX 27 kb)

Additional file 4: **Table S3.** DEGs identified in liver of E14 embryos between the fat and lean lines. (XLSX 125 kb)

Additional file 5: **Table S4.** DEGs identified in liver of E17 embryos between the fat and lean lines. (XLSX 138 kb)

Additional file 6: **Table S5.** DEGs identified in liver of E21 embryos between the fat and lean lines. (XLSX 31 kb)

Additional file 7: **Figure S2.** Protein profiling on liver tissues of chicken embryos. A total of 15 gels were assayed, and each gel contained three samples. Samples from the lean and fat chicken lines were labeled with Cy3 and Cy5, respectively. A pooled sample was used as the internal standard, labeled with Cy2. (DOC 4823 kb)

Additional file 8: **Figure S3.** Distribution of distinct tags. “Tags Containing N”, tags containing unknown bases; “Only adaptors”, reads containing only the adaptor sequence; “Copy Number < 2”, tags whose copy number is less than 2; “Clean tags”, tags remained after quality control and used for downstream analysis. L and F represent the lean and fat chicken lines, respectively. (DOC 176 kb)

Additional file 9: **Figure S4.** Proportion of clean tag numbers (> 96%). L and F represent the lean and fat chicken lines, respectively. (DOC 166 kb)

Additional file 10: **Figure S5.** Relationship of library size with percentage of genes identified. (DOC 64 kb)

Additional file 11: **Figure S6.** Number of antisense transcripts. L and F represent the lean and fat chicken lines, respectively. (DOC 144 kb)

Additional file 12: **Table S6.** Primer sequences used for qRT-PCR. (DOC 43 kb)

## Abbreviations

ACADL: Long-chain acyl-coenzyme A dehydrogenase
ACSBG2: Acyl-CoA synthetase bubblegum family member 2
AdipoR1: Adiponectin receptor 1
AFABP: Adipocyte fatty acid-binding protein
AFP: Abdominal fat percentage
ALDH7A1: Aldehyde dehydrogenase 7 family member A1
APOA4: Apolipoprotein A4
ApoA-I: Apolipoprotein A-I
ApoB: Apolipoprotein B
ApoE: Apolipoprotein E
BAP1: BRCA1 associated protein 1
CORO1C: Coronin-1C
DEGs: Differentially expressed genes
DEPs: Differentially expressed proteins
E12: Embryonic day 12
E14: Embryonic day 14
E17: Embryonic day 17
E21: Day 1 after hatch
E7: Embryonic day 7
FABP2: Fatty acid-binding protein, intestinal
GO: Gene ontology
HDL: High-density lipoprotein
HSPA9: Heat shock 70 kDa protein 9
HSPB1: Heat shock protein beta-1
IL8: Interleukin 8-like 2
ITPA: Inosine triphosphatase
KARS: Lysyl-tRNA synthetase
LBFABP: Liver basic fatty acids binding protein
LPL: Lipoprotein lipase
MTMR9: Myotubularin related protein 9
NDK: Nucleoside diphosphate kinase
NDUFB10: PREDICTED: NADH dehydrogenase [ubiquinone] 1 beta subcomplex subunit 10
NEAUHLF: Northeast Agricultural University broiler lines
OVAL: Ovalbumin
PPARγ: Peroxisome proliferator-activated receptor gamma
SDHA: Succinate dehydrogenase [ubiquinone] flavoprotein subunit, mitochondrial
SULT: Sulfotransferase
TBP: TATA box-binding protein
TCA cycle: Citrate cycle
TMEM79: Transmembrane protein 79
TXNDC5: Thioredoxin domain-containing protein 5
VLDL: Very low-density lipoprotein
XPO5: Exportin 5
YARS: Tyrosyl-tRNA synthetase
