# Supplementary material for: Embryonic transcriptome and proteome analyses on hepatic lipid metabolism in chickens divergently selected for abdominal fat content
Source: BMC Genomics. 2018 May 23;19:384. doi: 10.1186/s12864-018-4776-9 (PMC5966864; doi:10.1186/s12864-018-4776-9)

Additional file 1. Number of genes expressed and novel transcripts. L and F represent the lean and fat chicken lines, respectively.


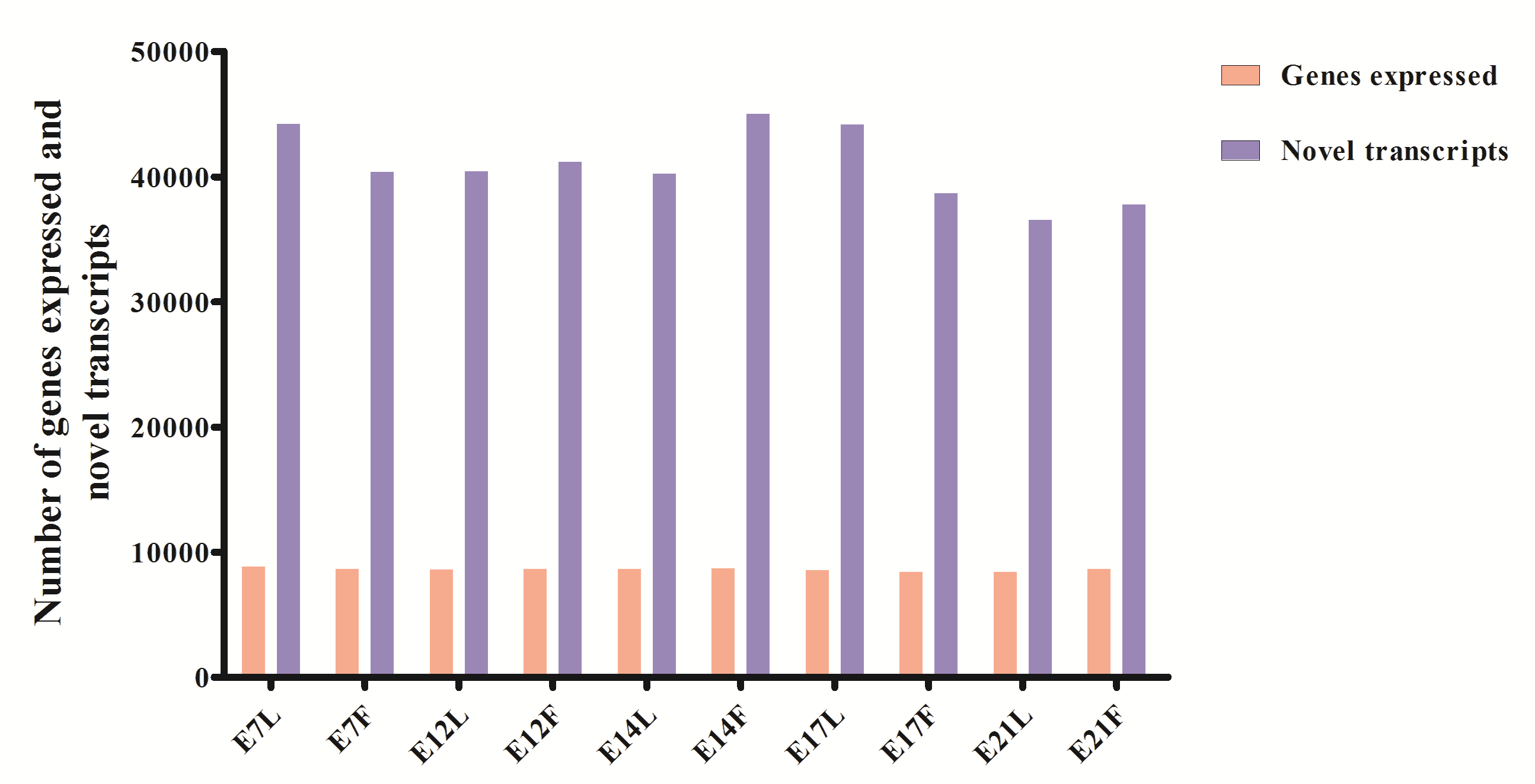

Supplement: Supplementary file 1 — Figure S1. Number of genes expressed and novel transcripts. L and F represent the lean and fat chicken lines, respectively. (DOC 177 kb) [file 12864_2018_4776_MOESM1_ESM.doc]
