# Supplementary material for: Embryonic transcriptome and proteome analyses on hepatic lipid metabolism in chickens divergently selected for abdominal fat content
Source: BMC Genomics. 2018 May 23;19:384. doi: 10.1186/s12864-018-4776-9 (PMC5966864; doi:10.1186/s12864-018-4776-9)
Supplement: Supplementary file 7 — Figure S2. Protein profiling on liver tissues of chicken embryos. A total of 15 gels were assayed, and each gel contained three samples. Samples from the lean and fat chicken lines were labeled with Cy3 and Cy5, respectively. A pooled sample was used as the internal standard, labeled with Cy2. (DOC 4823 kb) [file 12864_2018_4776_MOESM7_ESM.doc]

Additional file 7. Protein profiling on liver tissues of chicken embryos. A total of 15 gels were assayed, and each gel contained three samples. Samples from the lean and fat chicken lines were labeled with Cy3 and Cy5, respectively. A pooled sample was used as the internal standard, labeled with Cy2.

| **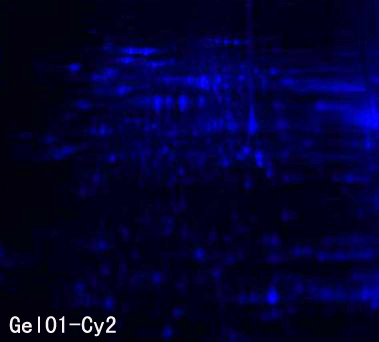** | **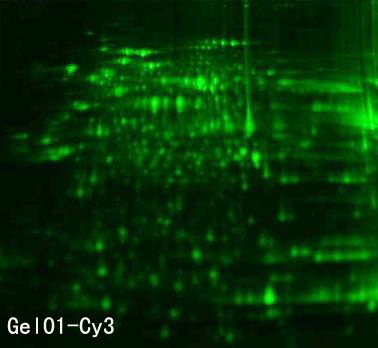** |
| --- | --- |
| **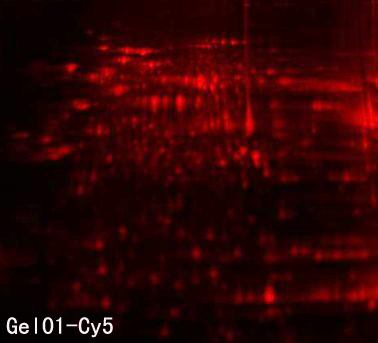** | **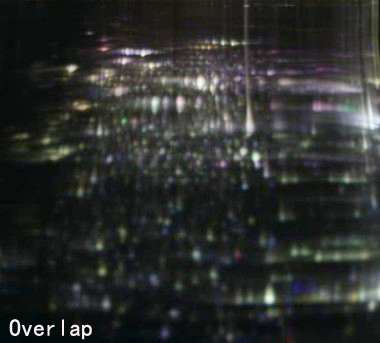** |
| **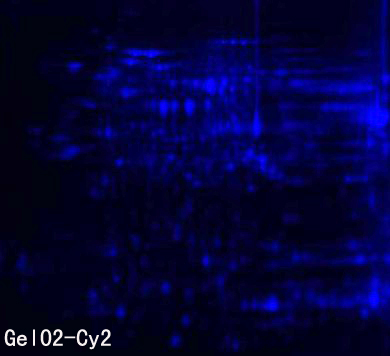** | **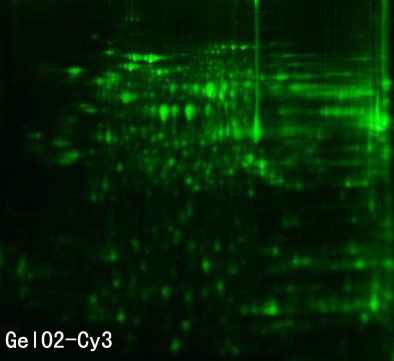** |
| **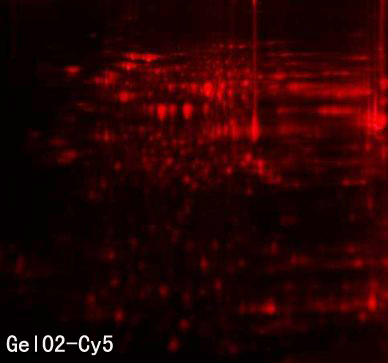** | **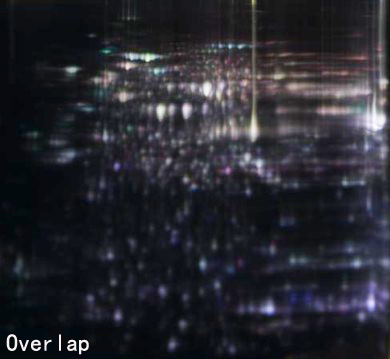** |
| **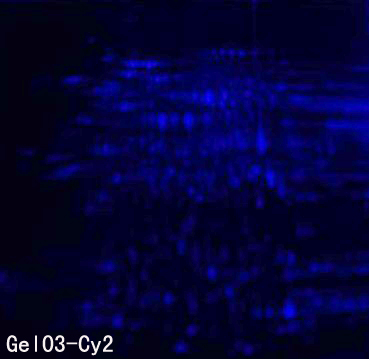** | **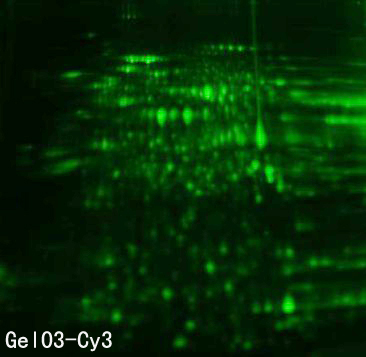** |
| **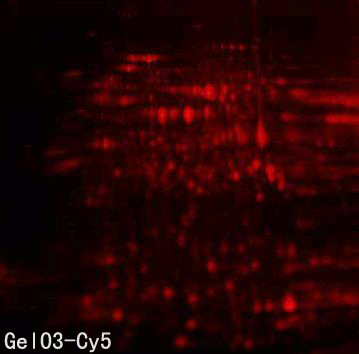** | **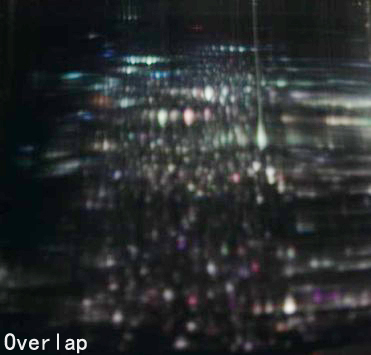** |
| **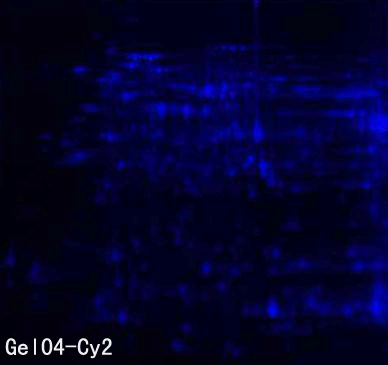** | **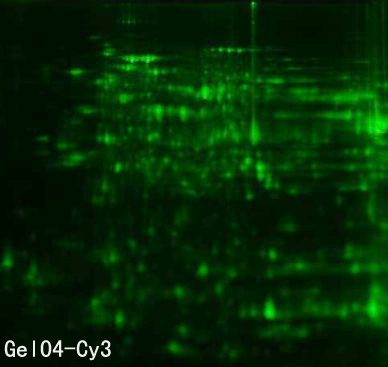** |
| **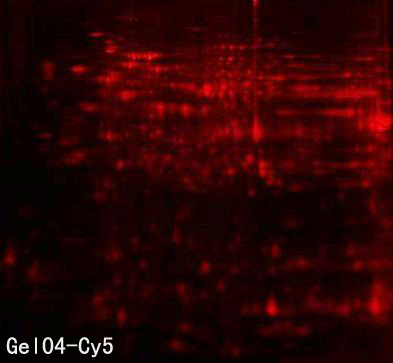** | **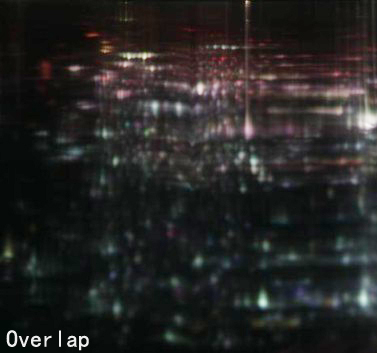** |
| **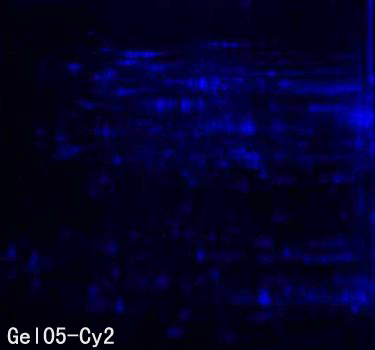** | **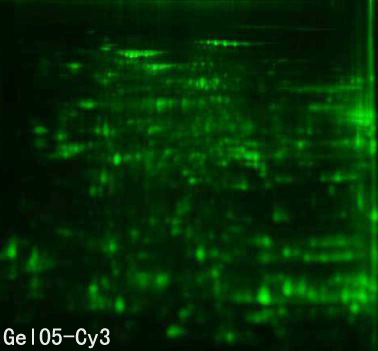** |
| **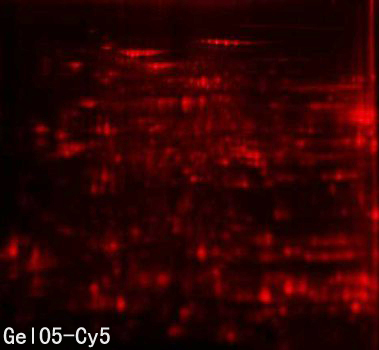** | **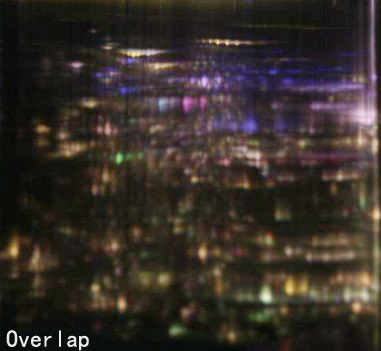** |
| **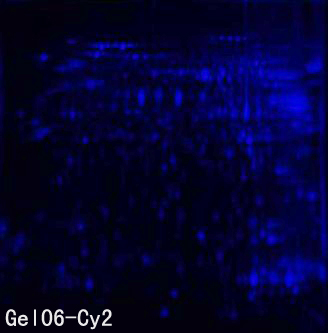** | **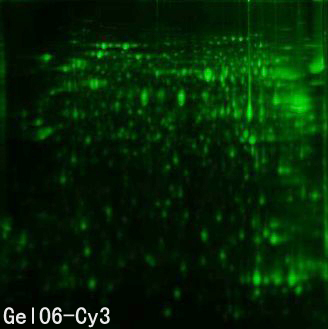** |
| **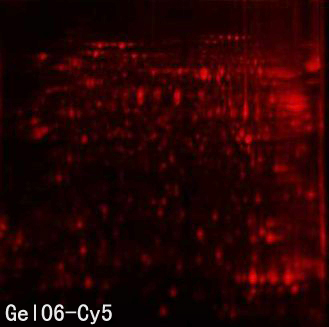** | **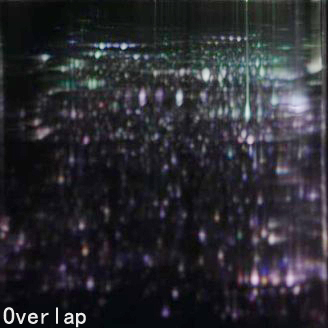** |
| **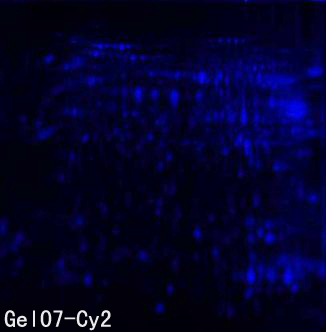** | **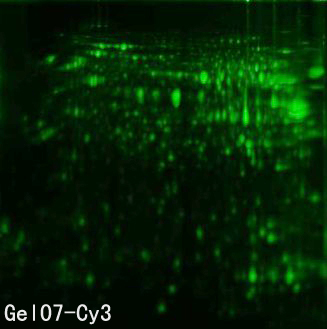** |
| **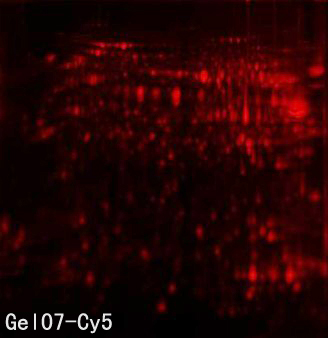** | **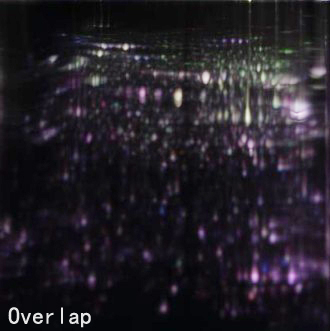** |
| **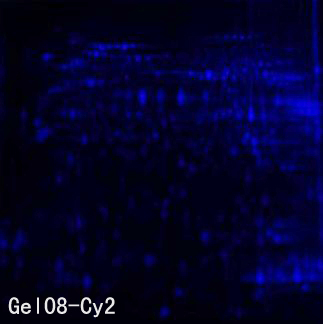** | **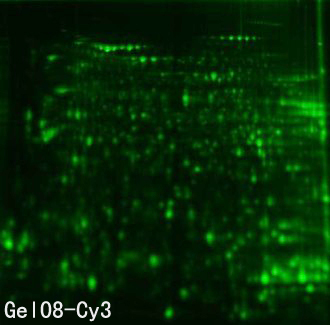** |
| **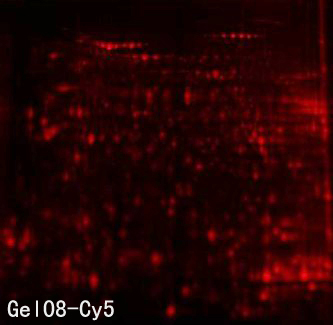** | **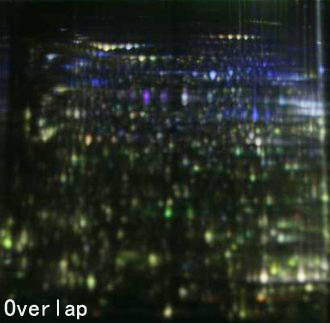** |
| **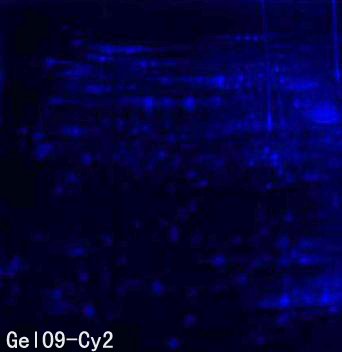** | **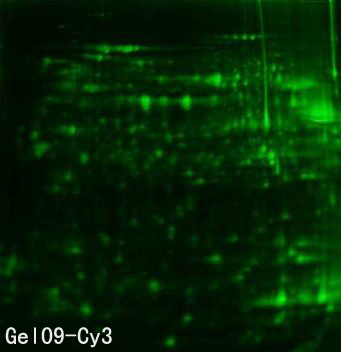** |
| **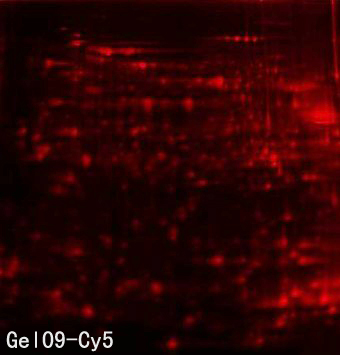** | **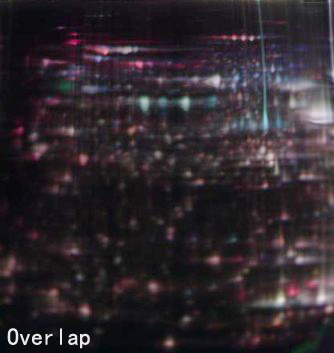** |
| **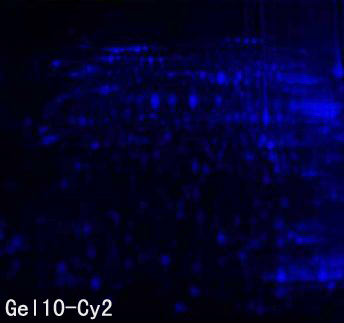** | **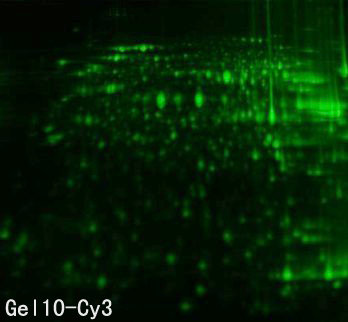** |
| **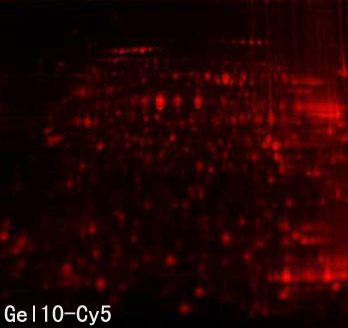** | **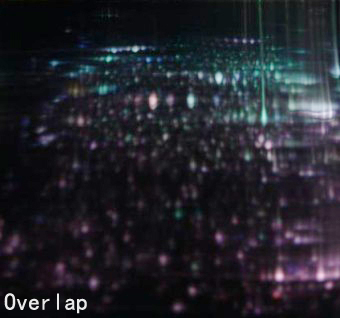** |
| **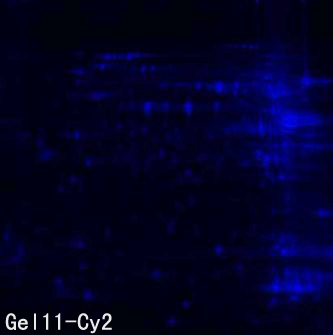** | **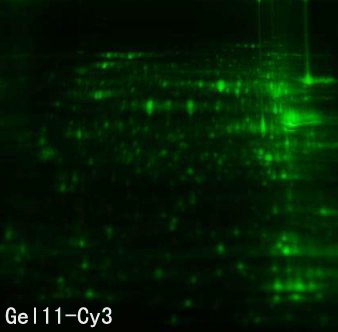** |
| **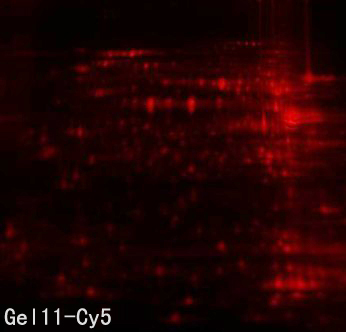** | **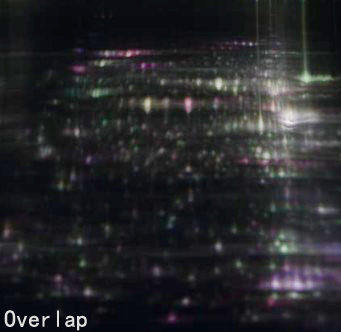** |
| **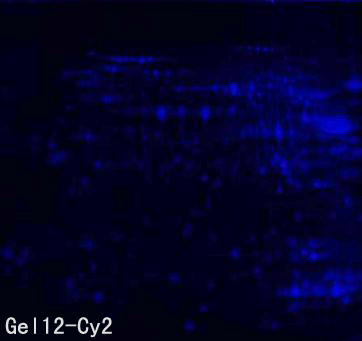** | **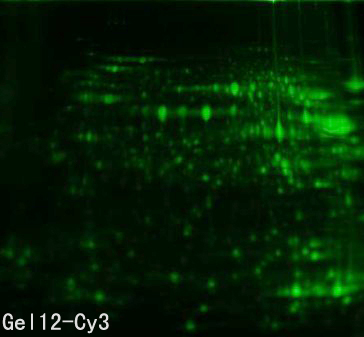** |
| **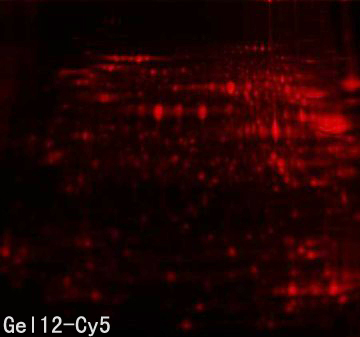** | **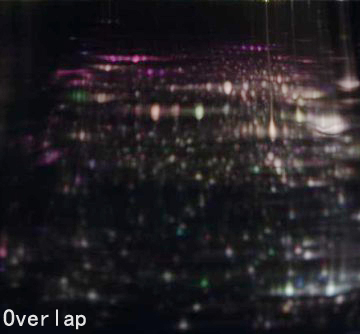** |
| **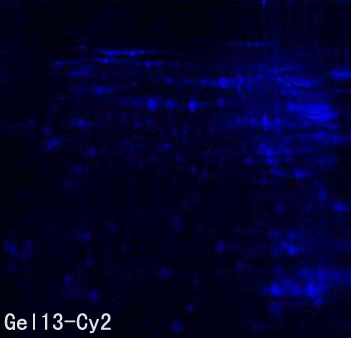** | **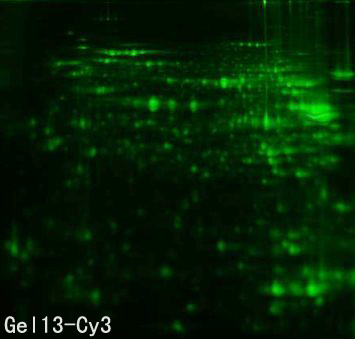** |
| **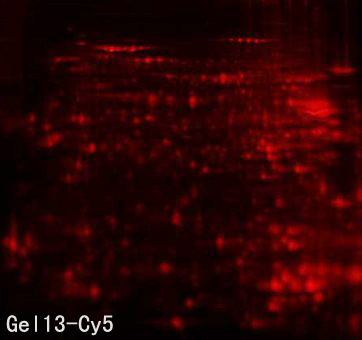** | **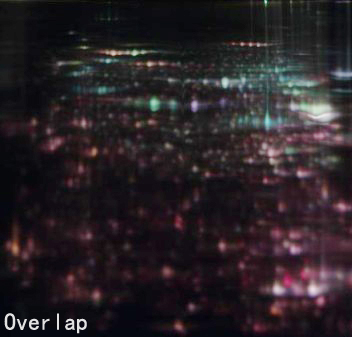** |
| **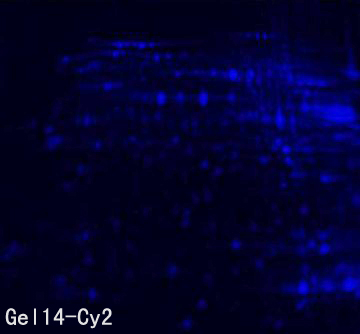** | **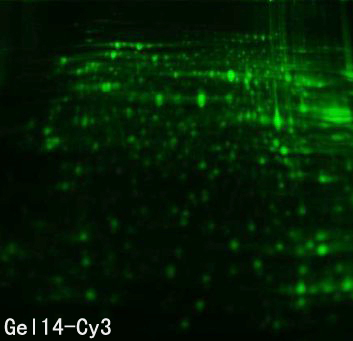** |
| **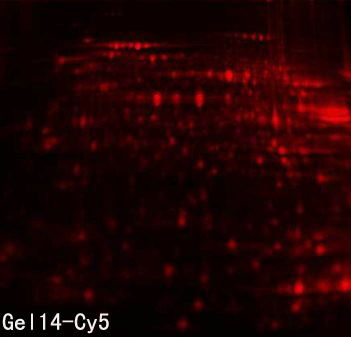** | **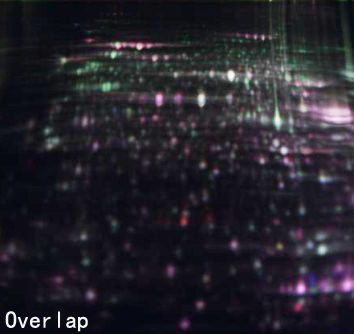** |
| **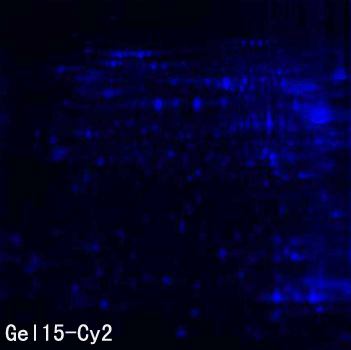** | **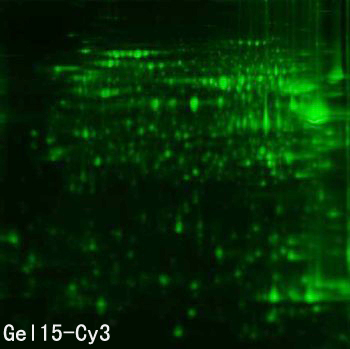** |
| **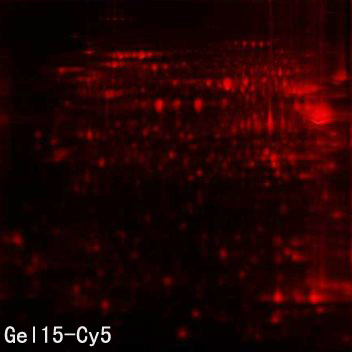** | **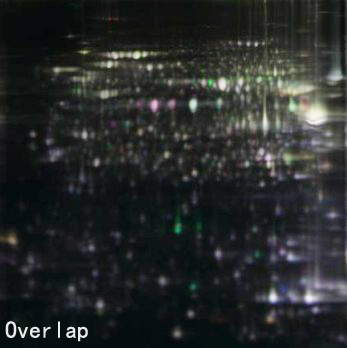** |
