# Supplementary material for: Embryonic transcriptome and proteome analyses on hepatic lipid metabolism in chickens divergently selected for abdominal fat content
Source: BMC Genomics. 2018 May 23;19:384. doi: 10.1186/s12864-018-4776-9 (PMC5966864; doi:10.1186/s12864-018-4776-9)

Additional file 8. Distribution of distinct tags. “Tags Containing N”, tags containing unknown bases; “Only adaptors”, reads containing only the adaptor sequence; “Copy Number < 2”, tags whose copy number is less than 2; “Clean tags”, tags remained after quality control and used for downstream analysis. L and F represent the lean and fat chicken lines, respectively.


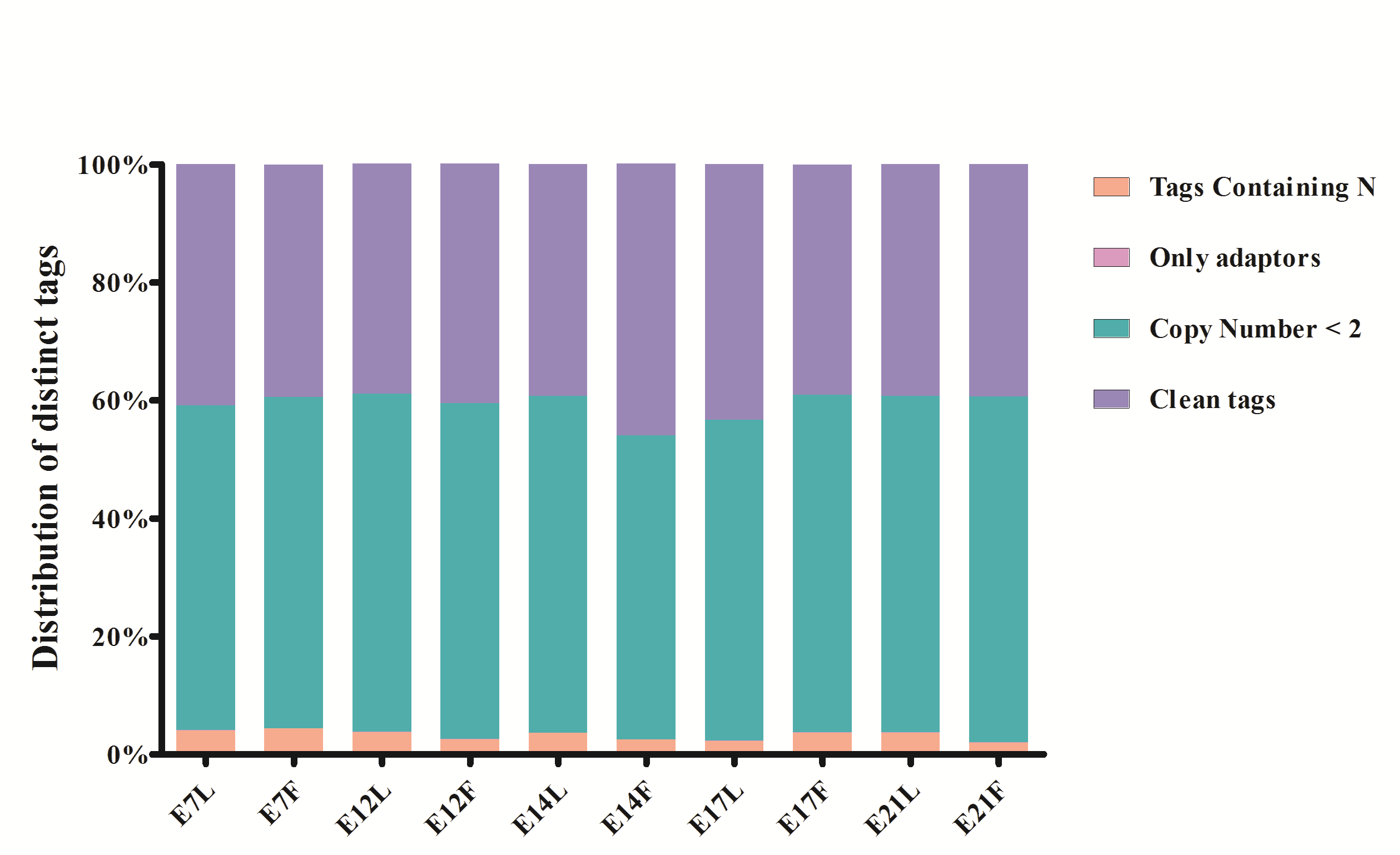

Supplement: Supplementary file 8 — Figure S3. Distribution of distinct tags. “Tags Containing N”, tags containing unknown bases; “Only adaptors”, reads containing only the adaptor sequence; “Copy Number < 2”, tags whose copy number is less than 2; “Clean tags”, tags remained after quality control and used for downstream analysis. L and F represent the lean and fat chicken lines, respectively. (DOC 176 kb) [file 12864_2018_4776_MOESM8_ESM.doc]
