# Supplementary material for: Embryonic transcriptome and proteome analyses on hepatic lipid metabolism in chickens divergently selected for abdominal fat content
Source: BMC Genomics. 2018 May 23;19:384. doi: 10.1186/s12864-018-4776-9 (PMC5966864; doi:10.1186/s12864-018-4776-9)
Supplement: Supplementary file 10 — Figure S5. Relationship of library size with percentage of genes identified. (DOC 64 kb) [file 12864_2018_4776_MOESM10_ESM.doc]

Additional file 10. Relationship of library size with the percentage of genes identified.
