# Supplementary material for: Embryonic transcriptome and proteome analyses on hepatic lipid metabolism in chickens divergently selected for abdominal fat content
Source: BMC Genomics. 2018 May 23;19:384. doi: 10.1186/s12864-018-4776-9 (PMC5966864; doi:10.1186/s12864-018-4776-9)

Additional file 11. Number of antisense transcripts. L and F represent the lean and fat chicken lines, respectively.


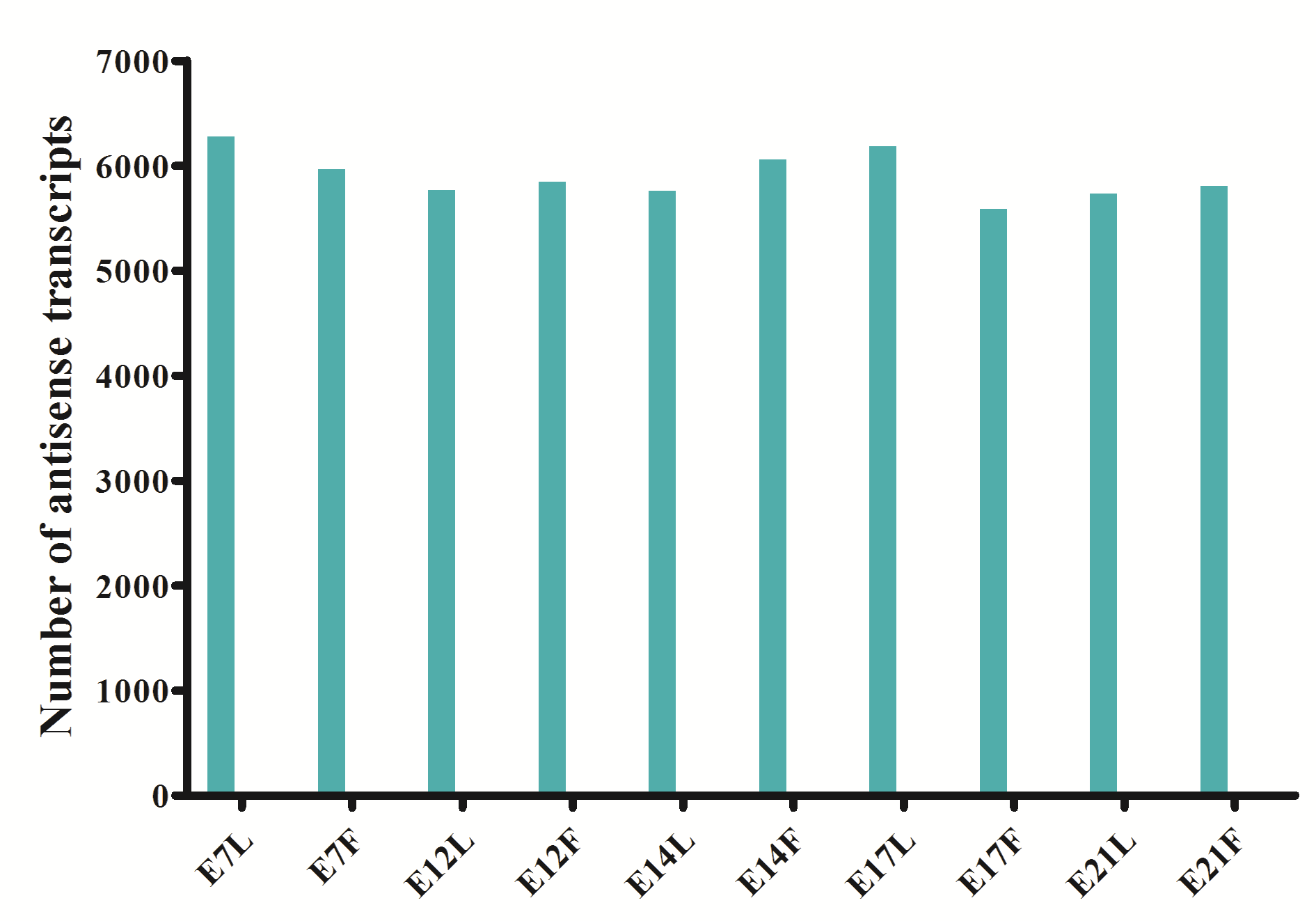

Supplement: Supplementary file 11 — Figure S6. Number of antisense transcripts. L and F represent the lean and fat chicken lines, respectively. (DOC 144 kb) [file 12864_2018_4776_MOESM11_ESM.doc]
