# Supplementary material for: Embryonic transcriptome and proteome analyses on hepatic lipid metabolism in chickens divergently selected for abdominal fat content
Source: BMC Genomics. 2018 May 23;19:384. doi: 10.1186/s12864-018-4776-9 (PMC5966864; doi:10.1186/s12864-018-4776-9)
Supplement: Supplementary file 12 — Table S6. Primer sequences used for qRT-PCR. (DOC 43 kb) [file 12864_2018_4776_MOESM12_ESM.doc]

Additional file 12. Primer sequences used for qRT-PCR.

| Gene | Accession Number | Primer sequence (5ʹ to 3ʹ) |
| --- | --- | --- |
| *LOC777109* | XM_001236580 | F: TCCATGCTGACCCCATAACC  R: CTCCTCTTCCTCCTCCCACAT |
| *LOC769366* | XM_001232626 | F: CATAGGCAGTGCGGAGGAGA  R: CCAGTTGGTGAAGGCTGTGAC |
| *APOA4* | NM_204938 | F: CACTCAGGATGTCGCCTAAAGC  R: GCAGTTGGTCCACGGTCTCC |
| *MTMR9* | NM_001031101 | F: TCTATCTGCCCTTCCTACCCTCC  R: GCGTTTCCCGTTTGTGCCT |
| *ACSBG2* | NM_001012846 | F: CCTGGTAGACACCCTGCGTG  R: CCTAGCCAAGCGGAAGTTCAT |
| *IL8* | NM_205498 | F:CAGAAAACAAGCCAAACACTCC  R:CCCATCTTTACCAGCGTCCTA |
| *TMEM79* | XM_424371 | F: CCAGTCCGTCCACCTCTTCATC  R: CAGCCAGTAGGTCAGCCGAGA |
| *KRT75* | NM_001001314 | F: ATCGACAAGGTCCGGTTCC  R: AGGGGCTCAATGTTGTTTCTG |
| *HSPA9* | NM_001006147 | F: CAGAGACAGGCGACGAAAGAT  R: GCCACCACCCAAGTCATAAAC |
| *BAP1* | NM_001030590 | F: AGAGCCCACCCACCAAATCAA  R: TGCTTTACCTGCCCCTTCCTT |
| *XPO5* | XM_419501 | F: ATTTCTACACGGTTGAGGGTCTT  R: ACACGGAGCATAGGTCGCA |
| *ALDH7A1* | XM_424422 | F: ACTGGAACTGGGAGGAAACAAT  R: AGCAGCAAACAAAGTAGATGGG |
| *KARS* | NM_001030583 | F: ATCGCATTCATCGGGGACTA  R: CTTTTGCCTTAGCCTGGTCTTC |
| *SDHA* | NM_001277398 | F: GTTTGTCCGAGGCTGGGTTTA  R: GCTGGGGCTTGCTCAGTCAT |
| *YARS* | [NM_001006314](https://www.ncbi.nlm.nih.gov/nuccore/NM_001006314.1) | F: CTTGACCCCATCAGGGAGAAAT  R: GGCTCAGGCTCACCCACATC |
| *CORO1C* | NM_001277869 | F: GATGCCTCAGACACTCAAAACGA  R: ATCTTCGCCATCTGCTGTTCC |
| *TXNDC5* | NM_001006374 | F: GCTCTGAGTTTGGCGTCCG  R: CATACATTCCCTGCTTAGGTTCG |
| *SULT* | NM_205060 | F: TTCTTTGGGATTTCCCTGACT  R: GTTTCGTTCTTGCTATGCGTT |
| *ApoA-I* | NM_205525 | F: GTGACCCTCGCTGTGCTCTT  R: CACTCAGCGTGTCCAGGTTGT |
| *HSPB1* | NM_205290 | F: TGCTTCACCCGCAAATACACC  R: TCTCGGATGACTGGATGGCTG |
| *NDK* | NM_205047 | F: AAGACCTCCTGAAGCAGCATTACA  R: CACCCTGCCTGTTTTCACCAC |
| *ITPA* | NM_001271930 | F: GTAACGGGCAACGCTAAGAA  R: TGGTACTCGGGCAGGTCAA |
| *NDUFB10* | XM_414844 | F: CAGTGGAGGAGGGACAGGTTG  R: GCATTTCCGTGGACACCAAGAT |
| *FABP2* | NM_001007923 | F: ATGGGCGTGAATGTGATGAA  R: GATGGTACGGAAGTTGCTTGAT |
